# Supplementary material for: Single-base 2′OMe-modified LNA and MOE gapmers selectively silence ACVR1R206H in fibrodysplasia ossificans progressiva
Source: Mol Ther Nucleic Acids. 2026 Apr 16;37(2):102937. doi: 10.1016/j.omtn.2026.102937 (PMC13276142; doi:10.1016/j.omtn.2026.102937)
Supplement: Document S1. Figures S1–S6 and Table S1 [file mmc1.pdf]

OMTN, Volume 37

Supplemental information

Single-base 2′OMe-modified LNA and MOE gapmers selectively silence *ACVR1*^{R206H} in fibrodysplasia ossificans progressiva

Saeed Anwar, Sarah Hay, Hidenori Moriyama, Farin Mir, Rika Maruyama, and Toshifumi Yokota

Table S1: Predicted off-target candidates for lead antisense gapmers. Potential off-target transcripts were identified at the web interface of GGGenome server (https://gggenome.dbcls.jp), allowing for up to 2 mismatches/indels for MOE gapmers and 1 for LNA gapmers. Alignments show the target gene sequence relative to the designated gapmer sequence. Bold indicates base mismatches, dash (–) denotes deletions, and underline denotes insertions. We searched against the human RefSeq RNA database (release 230, May 2025), for both the sense and antisense strands. *ACVR1*/*ACVR1*^{R206H} is excluded from this list.

Gene	Sequence	Number of mismatches
**MOE3r** TGG CTC ACC AGA TTA CAC TG		
ANP32B	TGG CT- ACC AG- TTA CAC TG	2
**MOE3s** TGG CTC ACC AGC TTA CAC TG		
ANP32B	TGG CT- ACC AG- TTA CAC TG	2
**LNA16r** CAG TGG CTC ACC AGA T		
TEC	CAG TGG CTC -CC AGA T	1
TENM1	CAG -GG CTC ACC AGA T	1
CD163L1	CAG -GG CTC ACC AGA T	1
MTERF4	CAG TGG CTC -CC AGA T	1
IRF2BP1	CAG TGG CTC **G**CC AGA T	1
NFATC3	CAG TGG CT**G** ACC AGA T	1
NCF4	CAG TGG CTC -CC AGA T	1
PCDH17	ATC TGG TGA G-C ACT G	1
SF3B3	ATC TGG TGA G-C ACT G	1
**LNA16s** CAG TGG CTC ACC AGC T		
CEMP1	CAG -GG CTC ACC AGC T	1
PIGG	CA**C** TGG CTC ACC AGC T	1
EXTL3	CAG -GG CTC ACC AGC T	1
ZNHIT6	CAG TGG CTC A-C AGC T	1
PRICKLE1	CAG TGG C-C ACC AGC T	1
FERMT2	CAG TGG C**A**C ACC AGC T	1
BCL11B	CAG -GG CTC ACC AGC T	1
CD163L1	CAG -GG CTC ACC AGC T	1
MTERF4	CAG TGG CTC ACC AG**G** T	1
ACSF3	C**T**G TGG CTC ACC AGC T	1
HEBP2	CAG TGG CTC A-C AGC T	1
CYP561D1	CAG TGG CTC -CC AGC T	1
STRN	AGC TGG TGA GCC -CT G	1
GALNT14	AGC -GG TGA GCC ACT G	1
TNK2	AGC T-G TGA GCC ACT G	1
ADGRA3	AGC T-G TGA GCC ACT G	1
GALNT17	AGC T-G TGA GCC ACT G	1
CAMTA1	AGC TG**C** TGA GCC ACT G	1
TACC2	AGC TGG TGA GCC CAC TG	1
A1CF	AGC TGG **A**GA GCC ACT G	1
SPPL3	AGC T-G TGA GCC ACT G	1
ESPL1	AGC TGG TG- GCC ACT G	1
AMDHD2	AGC TGG TGA GCC -CT G	1
ATAD2B	AGC T**T**G TGA GCC ACT G	1
ASIC4	AGC TGG TG**G** GCC ACT G	1
ITPKB	AGC TGG -GA GCC ACT G	1
AANAT	AGC -GG TGA GCC ACT G	1
PCDHA9	AGC T-G TGA GCC ACT G	1
NPLOC4	AGC T-G TGA GCC ACT G	1
RPL15	AGC TGG TGA GCC A**G**T G	1
HOGA1	AGC T-G TGA GCC ACT G	1
KIF4B	AGC TGG TGA GCA CAC TG	1
PRDM16	AGC TGG TG- GCC ACT G	1

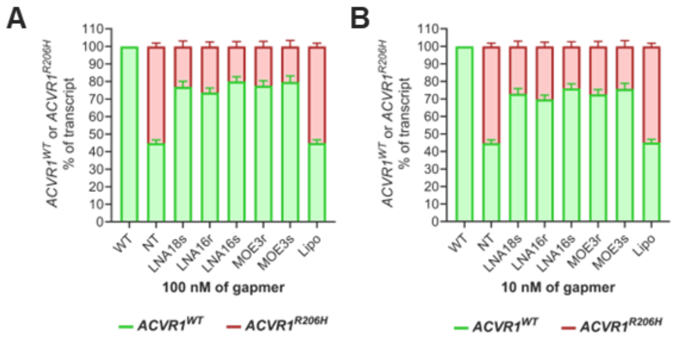

Figure S1. Gapmer treatment increases *ACVR1*^{WT} allelic contribution while reducing *ACVR1*^{R206H} expression in FOP patient-derived fibroblasts.

(**A–B**) Allelic composition of total *ACVR1* transcripts following treatment with 100 nM (**A**) or 10 nM (**B**) gapmers. Green and red bars represent *ACVR1*^{WT} and *ACVR1*^{R206H} transcript fractions, respectively. Data are presented as the mean ± standard error of the mean for the fractions calculated from all replicates. NT, non-treated, Lipo, Lipofectamine 3000 (a commercially available transfection reagent)

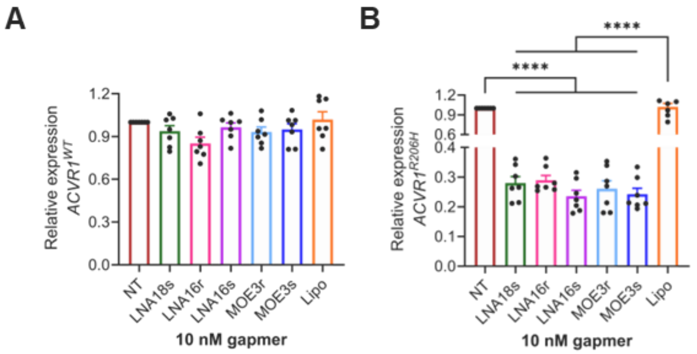

Figure S2. Selective suppression of *ACVR1^{R206H}* in C2C12 cells expressing V5-tagged constructs.

(**A–B**) RT-qPCR quantification of *ACVR1^{WT}* (**A**) and *ACVR1^{R206H}* (**B**) transcripts in C2C12 cells transfected with plasmids encoding V5-tagged *ACVR1^{WT}* and *ACVR1^{R206H}* and treated with 10 nM gapmers for 48 hours. Statistics, one-way ANOVA with Tukey's multiple comparisons test; ****p<0.0001. Data are represented as mean ± standard error of the mean. Each black dot represents an individual biological replicate. NT, non-treated, Lipo, Lipofectamine 3000 (a commercially available transfection reagent).

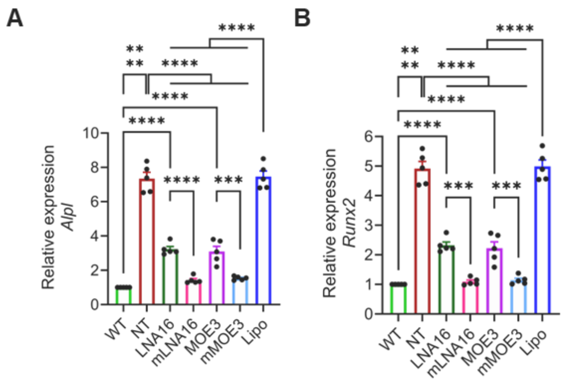

Figure S3. 2′OMe-modified gapmers suppress undesired osteogenic gene expression more effectively than unmodified counterparts.

(**A–B**) RT-qPCR quantification of osteogenic markers *Alpl* (**A**) and *Runx2* (**B**) in C2C12 cells transfected with V5-tagged $ACVR1^{WT}$ or $ACVR1^{R206H}$ and treated with 10 nM gapmers. Data collected 48 hours post-treatment. Statistics, one-way ANOVA with Tukey's multiple comparisons test; ****p<0.0001. Data are represented as mean ± standard error of the mean. Each black dot represents an individual biological replicate. NT, non-treated, Lipo, Lipofectamine 3000 (a commercially available transfection reagent).

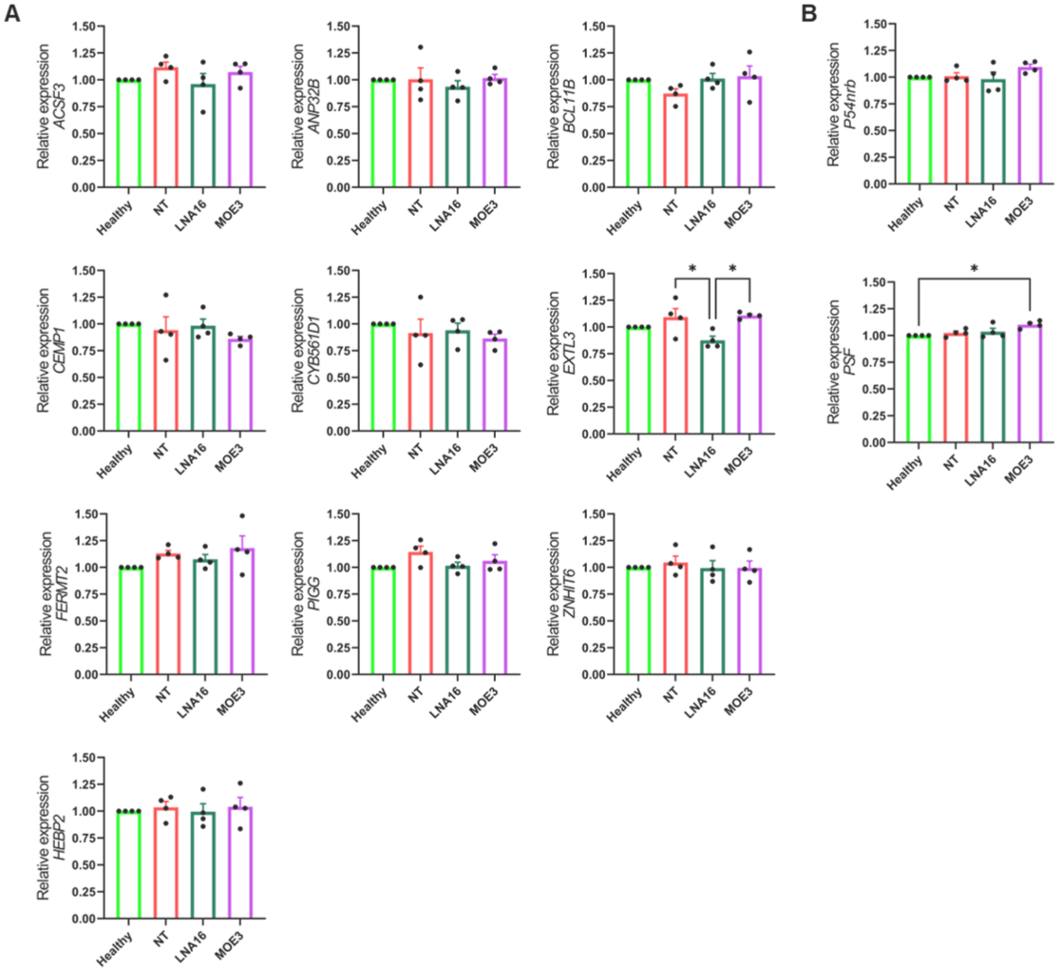

Figure S4. Gapmer treatment does not significantly alter the expression of predicted off-target genes or known ASO-sensitive genes.

(**A–B**) RT-qPCR assessment of the expression of (**A**) predicted off-target candidate genes and (**B**) paraspeckle genes known to be broadly affected by ASO treatments. FOP patient-derived fibroblasts were treated with 10 nM of LNA16 or MOE3 and RNA was harvested 48 hours post-treatment. Each black dot represents an individual biological replicate. Data are presented as the mean ± standard error of the mean. Statistics, one-way ANOVA with Tukey's multiple comparisons test; **p*<0.05.

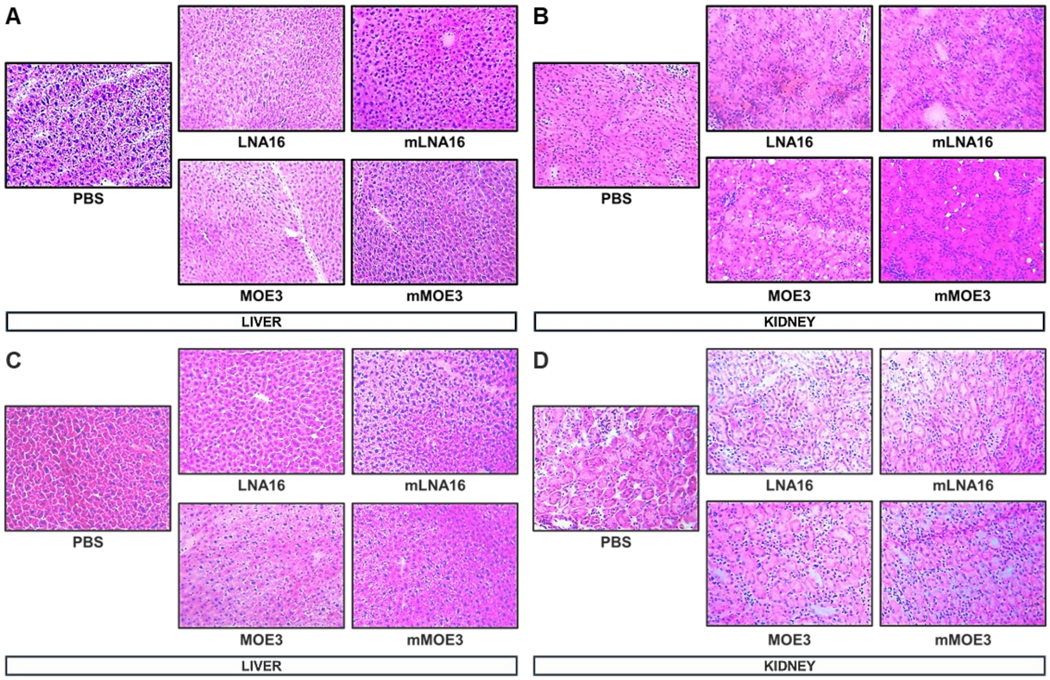

Figure S5: Histopathological analysis of liver and kidney following gapmer administration.

(**A–D**) Representative hematoxylin and eosin-stained sections of (**A, C**) liver and (**B, D**) kidney tissues harvested (**A, B**) 3 days and (**C, D**) 15 days post-injection. At 3 days, liver sections from mice treated with unmodified gapmers, i.e., LNA16 and MOE3, exhibited mild/diffuse hepatocellular rarefaction and microvesicular vacuolation, with no significant evidence of signs of necrosis, apoptosis, or inflammatory infiltrates. Mice receiving 2′OMe-modified gapmers, i.e., mLNA16 and mMOE3, demonstrated largely preserved hepatic architecture. In the kidney at 3 days, unmodified gapmers resulted in some localized proximal tubular epithelial vacuolation and mild luminal dilation. Importantly, signs of acute tubular necrosis or cast formation were not visible. 2′OMe-modified gapmers markedly attenuated these proximal tubular changes. By day 15 post-treatment, tissue architecture across all gapmer-treated groups was almost indistinguishable from PBS controls, confirming that early minor morphological shifts represent a transient, adaptive clearance response rather than progressive toxicologic pathology. Scale bar: 100 μm.

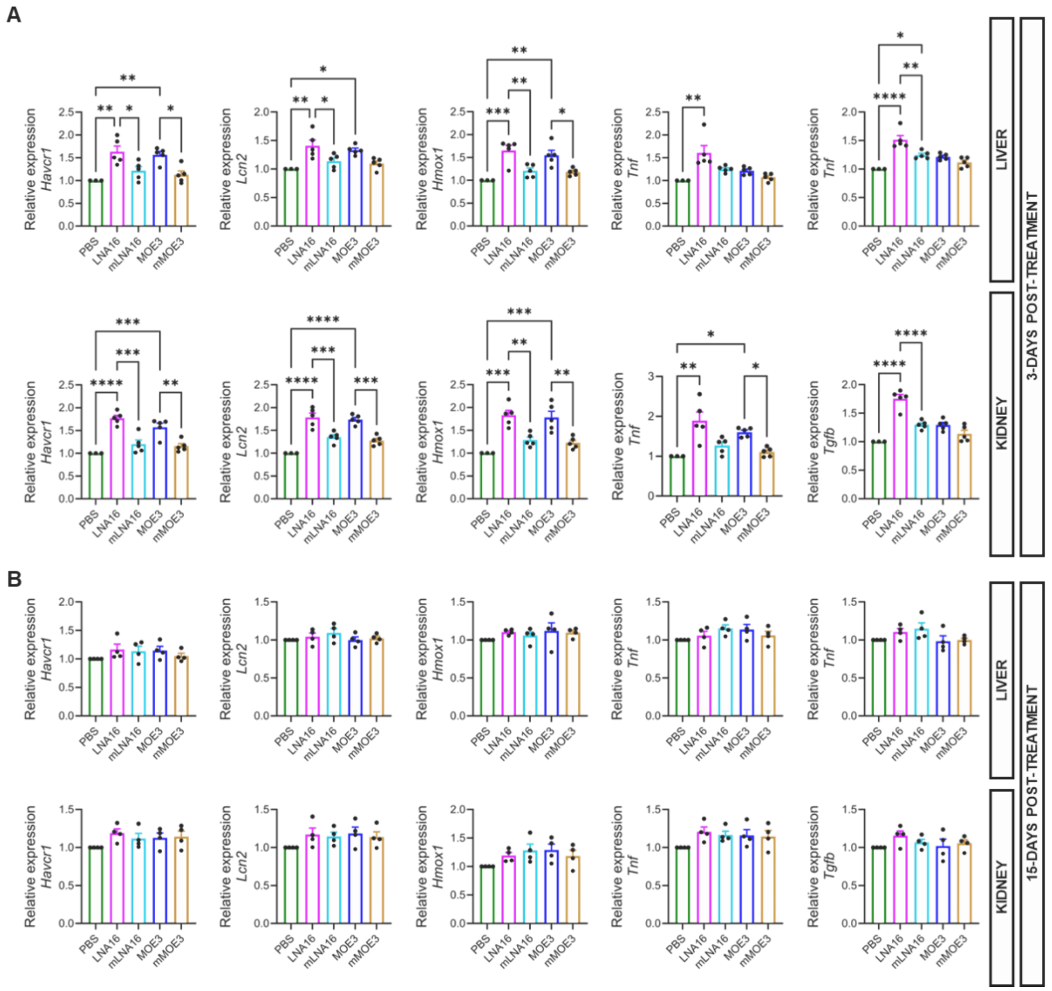

Figure S6: Hepatic and renal stress gene expression following gapmer administration.

(**A–B**) RT-qPCR quantification of a select panel of tissue stress markers, including *Havcr1*, *Lcn2*, *Hmox1*, *Tnf*, and *Tgfb* transcripts in liver and kidney tissues harvested at (**A**) 3 days and (**B**) 15 days post-treatment (11 mg/kg, retro-orbital). Wild-type B6 mice received retro-orbital (r.o.) administration of gapmers (11 mg/kg). Statistics, one-way ANOVA with Tukey's multiple comparisons test; *p<0.05, **p<0.01, ***p<0.001, ****p<0.0001. Data are represented as mean ± standard error of the mean. Each black dot represents an individual biological replicate.
