# Supplementary material for: Single-base 2′OMe-modified LNA and MOE gapmers selectively silence ACVR1R206H in fibrodysplasia ossificans progressiva
Source: Mol Ther Nucleic Acids. 2026 Apr 16;37(2):102937. doi: 10.1016/j.omtn.2026.102937 (PMC13276142; doi:10.1016/j.omtn.2026.102937)
Supplement: Document S2. Article plus supplemental information [file mmc2.pdf]

Molecular Therapy
Nucleic Acids
Original Article

Single-base 2′OMe-modified LNA and MOE gapmers selectively silence *ACVR1*^{R206H} in fibrodysplasia ossificans progressiva

Saeed Anwar,[1] Sarah Hay,[1,2] Hidenori Moriyama,[1] Farin Mir,[1,2] Rika Maruyama,[1] and Toshifumi Yokota[1,3,4,5,6]

[1]Department of Medical Genetics, Faculty of Medicine and Dentistry, University of Alberta, Edmonton, AB T6G 2H7, Canada; [2]Department of Biological Sciences, Faculty of Science, University of Alberta, Edmonton, AB T6G 2E9, Canada; [3]Cardiovascular Research Institute, Faculty of Medicine and Dentistry, University of Alberta, Edmonton, AB T6G 1C9, Canada; [4]Neuroscience and Mental Health Institute, Faculty of Medicine and Dentistry, University of Alberta, Edmonton, AB T6G 2E1, Canada; [5]Women and Children's Health Research Institute, Faculty of Medicine and Dentistry, University of Alberta, Edmonton, AB T6G 1C9, Canada; [6]The Friends of Garrett Cumming Research and Muscular Dystrophy Canada Endowed Research Chair and the Henri M. Toupin Chair in Neurological Science, Edmonton, AB T6G 2H7, Canada

Fibrodysplasia ossificans progressiva (FOP) is an ultra-rare disorder caused by gain-of-function mutations in *ACVR1*, most commonly c.617G>A (R206H), leading to progressive heterotopic ossification. In this study, we developed novel antisense gapmers selectively targeting the mutant *ACVR1*^{R206H} transcript while sparing the wild-type allele. We engineered locked nucleic acid (LNA) and 2′-O-methoxyethyl (MOE) gapmers incorporating a single 2′-O-methyl (2′OMe) modification at gap position 2. This is hypothesized to synergize with the wild-type sequence mismatch to restrict RNase H1 cleavage, limiting wild-type degradation while preserving mutant target engagement. In FOP patient-derived fibroblasts carrying the endogenous *ACVR1*^{R206H} mutation and in murine-derived C2C12 cells ectopically expressing *ACVR1*^{R206H} constructs, 2′OMe-modified gapmers demonstrated robust and preferential suppression of *ACVR1*^{R206H} at both RNA and protein levels. Gapmer treatment also reduced osteogenic differentiation, as shown by decreased alkaline phosphatase and Alizarin Red S staining, and lower expression of osteogenic markers. In wild-type mice, 2′OMe modification was associated with higher apparent gapmer levels in skeletal muscle and tendon and lower hepatic and renal stress marker readouts. These findings provide preliminary proof-of-concept that a single-base chemical modification can modulate allele selectivity and biodistribution of gapmers targeting *ACVR1*^{R206H}. Further studies in disease-relevant FOP models will be needed to establish therapeutic efficacy and long-term safety.

INTRODUCTION

Fibrodysplasia ossificans progressiva (FOP; OMIM #135100), commonly known as stone man syndrome, is an ultra-rare and debilitating genetic disorder characterized by progressive heterotopic ossification (HO), where soft tissues, including skeletal muscles, tendons, ligaments, and fascia, are pathologically transformed into bone through endochondral ossification.[1–4] FOP is the only known disorder in which one tissue type is replaced by another, providing a unique model to study cellular fate dysregulation and tissue homeostasis.[2–4] In affected individuals, HO typically manifests in childhood or early adulthood, often triggered by minor trauma, surgical interventions, intramuscular injections, viral infections, or inflammation.[4,5] Beyond HO, FOP patients experience additional complications, e.g., impaired speech, swallowing difficulties, and respiratory dysfunction, contributing to chronic pain, significant morbidity, and premature mortality.[4,6]

FOP is caused by heterozygous activating mutations in the type I bone morphogenetic protein (BMP) receptor ACVR1 (activin A receptor type I, also known as activin receptor-like kinase-2 or ALK2), a key component of BMP signaling pathways.[4,7,8] The most common mutation, c.617G>A (R206H), leads to aberrant BMP signaling and an abnormal, gain-of-function response to activin A, driving ectopic bone formation. More than 90% of FOP cases are due to this recurrent *ACVR1*^{R206H} mutation within the glycine-serine domain.[4,7,8] Studies in animal models have identified fibro/adipogenic progenitors as the primary cellular source of HO in FOP.[9,10]

Despite advances in understanding the pathophysiology of FOP, no curative therapies currently exist. Surgical removal of heterotopic bone is contraindicated due to the risk of triggering aggressive postoperative bone regrowth.[11] Additionally, FOP patients also face anesthesia-related challenges, e.g., difficult intubation, pulmonary complications, and cardiac conduction abnormalities.[11,12] Current treatment strategies primarily focus on symptom management, including intermittent use of anti-inflammatory agents to suppress flare-up-induced inflammation.[11] However, even these interventions

Received 5 June 2025; accepted 15 April 2026;
https://doi.org/10.1016/j.omtn.2026.102937.

Correspondence: Toshifumi Yokota, PhD, University of Alberta, 8-31 Medical Sciences Building, Edmonton, AB T6G 2H7, Canada.
E-mail: toshifum@ualberta.ca

Published by Elsevier Inc. on behalf of The American Society of Gene and Cell Therapy.
This is an open access article under the CC BY license (http://creativecommons.org/licenses/by/4.0/).

may paradoxically exacerbate HO in some cases.[13] Various experimental therapies, including small-molecule inhibitors, monoclonal antibodies, and genetic therapies, are under evaluation.[4,14–17] These approaches aim to block activin A binding, inhibit ACVR1 kinase activity, target mTOR signaling, or activate retinoic acid receptor-γ (RARγ) to suppress chondrogenesis and endochondral ossification. Notably, an RARγ agonist, palovarotene (Sohonos), was recently approved by the US Food and Drug Administration (FDA) and Health Canada for FOP treatment, though its adverse effects limit its suitability for pediatric patients.[18]

Since FOP is driven by a hyperactive mutant ACVR1 receptor, allele-specific silencing of the *ACVR1*^{R206H} mutant transcript represents a promising therapeutic approach.[19,20] Such an approach could selectively target the mutant allele while preserving the normal function of the wild-type *ACVR1* allele. However, the challenge remains to design a therapy that avoids inhibiting the healthy allele, as *ACVR1* is essential for normal skeletal development and tissue homeostasis. Studies involving animal models suggest that deactivating *ACVR1* results in significant adverse effects.[21–23] Non-selective inhibition of ACVR1 would result in significant toxicity, as indicated by studies showing that complete deactivation of ACVR1 leads to detrimental effects in animal models.[4,21–23] Recent advances in antisense technology, including the use of gapmers and siRNA, have demonstrated the feasibility of selectively targeting the mutant *ACVR1* allele without affecting the wild-type allele.[19,24] This approach targets the mutated *ACVR1* allele, which causes HO through aberrant responsiveness to activin A, while sparing the healthy allele and preserving normal ACVR1 function. We recently reported an unprecedented strategy to selectively suppress the pathogenic *ACVR1*^{R206H} transcript using fully phosphorothioate (PS)-modified locked nucleic acid (LNA) gapmers.[24] In our proof-of-concept study, we have shown that our designed gapmers reduced *ACVR1*^{R206H} expression at RNA levels, while the healthy *ACVR1* allele was mostly unaffected in two FOP patient-derived fibroblast cell lines.[24] Also, the gapmers suppressed osteogenic differentiation induced by *ACVR1*^{R206H} and activin A. This novel antisense approach offers potential for therapeutic application in FOP.

Antisense gapmers are a class of oligonucleotides that induce RNA cleavage via RNase H recruitment.[25,26] Structurally, gapmers consist of a central DNA segment flanked by chemically modified RNA analogs, e.g., LNAs, 2′-O-methoxyethyl (MOE), or constrained ethyl (cEt), which enhance binding affinity and stability.[26,27] To improve pharmacokinetics and resistance to nucleases, gapmers typically incorporate PS modifications, replacing non-bridging oxygen atoms with sulfur atoms in the phosphate backbone.[28] While these chemical modifications generally enhance stability and efficacy, they can also elicit toxicity, including nephrotoxicity, hepatotoxicity, thrombocytopenia, and inflammation in preclinical and clinical settings.[26,29–39] Two primary mechanisms underlie these toxicities: off-target RNA cleavage due to sequence mismatches and non-specific interactions with cellular proteins, leading to unpredictable adverse effects.[34,35,38,39] Notably, small sequence or chemical modifications can profoundly influence the pharmacokinetics, toxicity, and therapeutic index of PS-ASOs.[29,31,36,40–43] More recent studies have shown that small modifications, e.g., a single 2′-O-methyl (2′OMe) substitution at the gap 2 position, can reduce off-target toxicity while maintaining potent antisense activity.[44,45] This modification improves the therapeutic index by decreasing unwanted protein-binding interactions, thereby enhancing the safety profile of antisense gapmers.

In this study, we investigate the efficacy and safety of LNA and MOE gapmers targeting the *ACVR1*^{R206H} allele, incorporating a single 2′OMe modification. We hypothesize that this modification improves the therapeutic index by enhancing allele-specific silencing while reducing toxicity. We systematically evaluate the efficacy of these modified gapmers in patient-derived fibroblasts and C2C12 cells, and assess their biodistribution, safety, and bioavailability in a mouse model. Our findings demonstrate that the 2′OMe-modified LNA and MOE gapmers selectively silence the *ACVR1*^{R206H} transcript while sparing the wild-type allele, and show improved tissue bioavailability, reduced renal and hepatic toxicity, and favorable safety profiles. This study provides the initial experimental evidence that a single-base 2′OMe modification can modulate allele selectivity and acute tissue distribution of gapmers in FOP models, warranting further preclinical evaluation in disease-relevant systems.

RESULTS

Gapmer treatment preferentially suppresses the pathogenic *ACVR1*^{R206H} expression while sparing the *ACVR1*^{WT} allele

We designed two novel allele-specific antisense gapmers targeting the *ACVR1* mRNA carrying the pathogenic c.617G>A (R206H) mutation (Figure 1A). These included an LNA-based gapmer, e.g., LNA16r, and an MOE-based gapmer, e.g., MOE3r. To enhance allele selectivity, we incorporated a T→G mismatch, which has previously been shown to improve allele discrimination in siRNA-based approaches targeting *ACVR1*^{R206H}.[19] These modified versions were denoted LNA16s and MOE3s, respectively. A previously validated LNA-based gapmer (LNA18s, previously referred to as AL-7s) was also included as a comparator.[24]

We first assessed the silencing efficiency of these gapmers in *ACVR1*$^{R206H/+}$ FOP patient-derived fibroblasts. Cells were treated at 100 and 10 nM concentrations, and total *ACVR1* expression was quantified using RT-qPCR 48 h post-transfection (Figure 1B). Compared to healthy fibroblasts, untreated FOP fibroblasts showed significantly elevated *ACVR1* transcript levels (Figures 1C and 1D). Gapmer treatments effectively reduced total *ACVR1* transcript by an average of 51.23 ± 1.11% at 100 nM and 49.36 ± 1.38% at 10 nM relative to non-treated FOP fibroblasts. Subsequent immunoblot analysis confirmed corresponding reductions in ACVR1 protein levels (Figures 1E–1G), with mean knockdown levels of 44.98 ± 1.9% and 42.71 ± 1.39% at 100 and 10 nM, respectively. We did not observe any significant difference in efficacy in reducing the expression of total ACVR1 between the gapmers with and without the mismatch. To determine allele-specific silencing activity, we

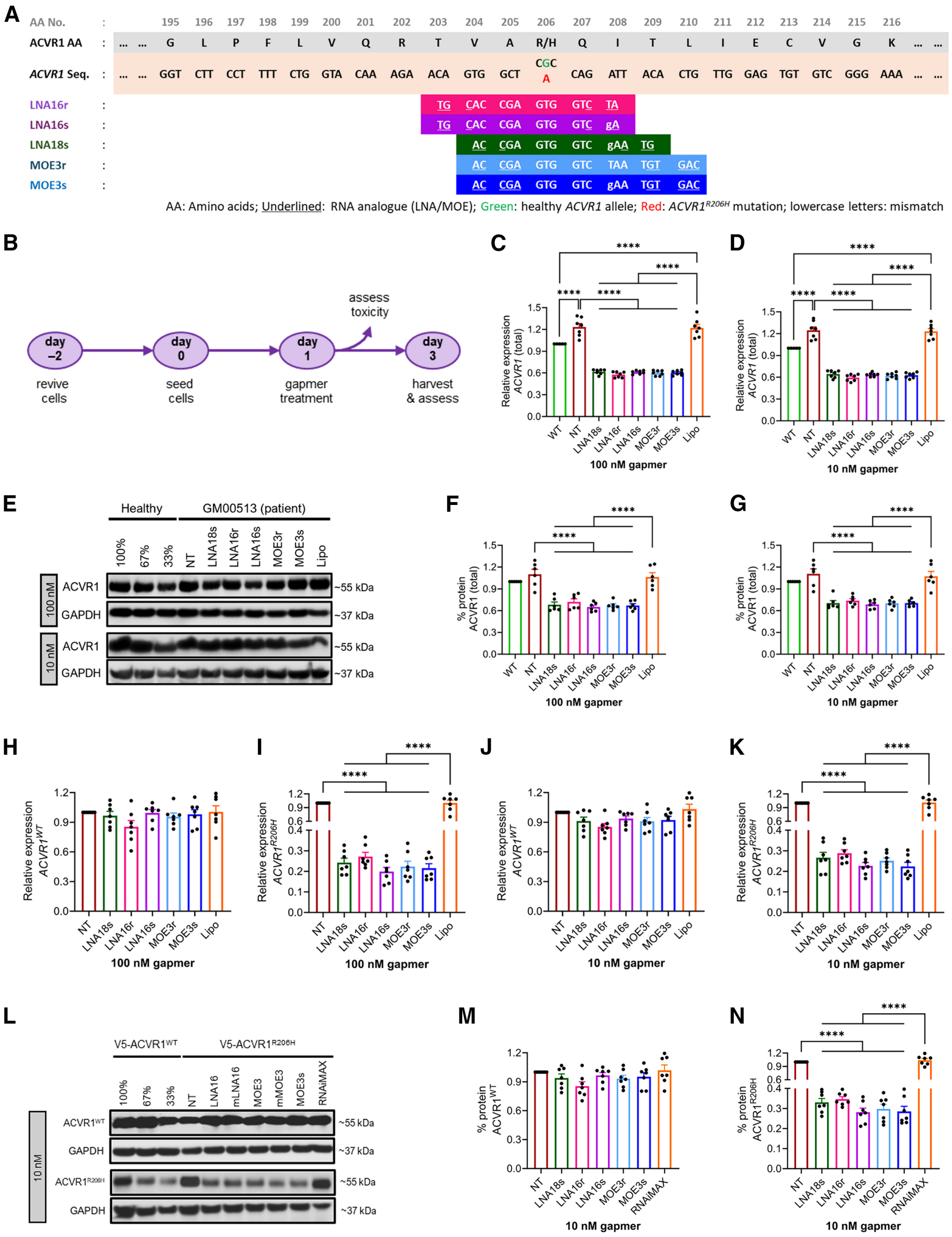

Figure 1. Preferential silencing of *ACVR1*^{R206H} in FOP patient-derived fibroblasts using LNA and MOE gapmers

(A) Sequence alignment showing LNA and MOE gapmer binding sites spanning the ACVR1 c.617G>A mutation (R206H). Mismatches are shown in lowercase; green: *ACVR1*^{WT} variant; red: *ACVR1*^{R206H} variant. (B) Schematic overview of the experimental timeline: day 2, cell revival; day 0, seeding; day 1, transfection; day 3, RNA/protein harvest. Toxicity assessments were performed at 4, 12, 24, and 48 h post-transfection. (C and D) RT-qPCR analysis of total ACVR1 mRNA levels following treatment with 100 nM (C) or 10 nM (D) gapmers. (E) Representative immunoblots showing total ACVR1 protein levels post-treatment with 100 and 10 nM gapmers. GAPDH served as a loading control. (F and G) Quantification of total ACVR1 protein abundance relative to healthy control cells at 100 nM (F) and 10 nM (G) using densitometric analysis of the immunoblot images (E). (H–K) Allele-specific RT-qPCR quantification of *ACVR1*^{WT} (H, J) and *ACVR1*^{R206H} (I, K) transcript levels following treatment with 100 nM (H, I) or 10 nM (J, K) gapmers. (L) Representative immunoblots of V5-tagged *ACVR1*^{WT} and *ACVR1*^{R206H} proteins expressed in C2C12 cells 48 h post-treatment with 10 nM gapmers, with GAPDH as a loading control. (M and N) Densitometric quantification of immunoblot images (L) showing V5-tagged *ACVR1*^{WT} (M) and *ACVR1*^{R206H} (N) protein levels in C2C12 cells relative to untreated controls. Statistics, one-way ANOVA with Tukey's multiple comparisons test; ****$p < 0.0001$. Data are represented as mean ± standard error of the mean (n = 5–7). NT, non-treated; Lipo, lipofectamine 3000 (a commercially available transfection reagent).

quantified *ACVR1*^{WT} and *ACVR1*^{R206H} transcripts using allele-specific RT-qPCR (Figures 1H–1K and S1). All tested gapmers significantly reduced *ACVR1*^{R206H} expression while largely sparing *ACVR1*^{WT} transcript, leading to an increased *ACVR1*^{WT}/*ACVR1*^{R206H} ratio (Figure S1). Among all candidates, LNA16s achieved the strongest mutant-allele specific knockdown, 77.38 ± 4.92% at 10 nM and 80.12 ± 5.31% at 100 nM (Figures 1I and 1K). Importantly, the differences in *ACVR1*^{R206H} suppression efficacy between 10 nM and 100 nM were minimal across all gapmers (mean Δ = 2.74%), supporting the use of lower doses for downstream applications.

To further evaluate specificity, we tested gapmers in C2C12 myoblasts ectopically expressing V5-tagged *ACVR1*^{WT} or *ACVR1*^{R206H} constructs. Consistent with our patient-derived fibroblast results, RT-qPCR confirmed that all gapmers preferentially suppressed *ACVR1*^{R206H} transcript, with minimal effects on *ACVR1*^{WT} (Figure S2). Immunoblotting corroborated these findings at the protein level; while total ACVR1^{WT} levels remained largely unchanged (Figure 1M), *ACVR1*^{R206H} protein abundance was significantly reduced across the board (Figure 1N), with LNA16s achieving the most potent *ACVR1*^{R206H} knockdown (71.86 ± 5.03%). Interestingly, among the LNA-based gapmers, those incorporating the T→G mismatch (i.e., LNA16s and LNA18s) consistently exhibited improved mutant allele discrimination without significantly affecting wild-type expression. For MOE-based gapmers; however, the presence (i.e., MOE3s) or absence (i.e., MOE3r) of the mismatch did not significantly alter allele selectivity, suggesting a context-dependent effect of mismatch engineering that may be influenced by scaffold chemistry or structural constraints.

Together, these results demonstrate that our designed gapmers effectively and preferentially silence the mutant *ACVR1*^{R206H} allele in FOP patient-derived fibroblasts and engineered myoblasts while largely sparing wild-type *ACVR1* expression. Because LNA16s and MOE3r exhibited the most optimal allelic discrimination ratios, they were chosen as the lead candidates for all subsequent modification and functional experiments. In addition, because target knockdown reached a near-plateau level at 10 nM, suggesting near-maximal RNase H saturation, this concentration was prioritized for most of the downstream functional and safety assessments to maximize the therapeutic index.

Single-base 2′OMe modification enhances allele selectivity and suppresses osteogenic differentiation *in vitro*

To improve the safety and selectivity of our lead gapmers, we incorporated a single 2′OMe modification at gap position 2 on the 5′ end of both LNA16 and MOE3. These modified gapmers, hereafter referred to as mLNA16 and mMOE3, were evaluated alongside their unmodified counterparts in FOP patient-derived fibroblasts and engineered C2C12 cells expressing V5-tagged *ACVR1*^{WT} or *ACVR1*^{R206H} constructs. We first assessed allele-selective knockdown in FOP patient-derived fibroblasts using allele-specific RT-qPCR (Figures 2A–2C). All gapmers selectively suppressed *ACVR1*^{R206H} with minimal effects on *ACVR1*^{WT}, but the modified versions exhibited enhanced allele discrimination. Both mLNA16 and mMOE3 increased the *ACVR1*^{WT}/*ACVR1*^{R206H} transcript ratio more effectively than their unmodified counterparts (Figures 2B and 2C), indicating improved selectivity conferred by the 2′OMe modification.

To validate these findings in an independent system, we repeated the analysis in C2C12 myoblasts overexpressing V5-tagged *ACVR1*^{WT} or *ACVR1*^{R206H}. RT-qPCR again confirmed preferential suppression of the mutant transcript with little impact on the wild-type allele (Figures 2D–2F), and the 2′OMe-modified gapmers showed enhanced suppression of *ACVR1*^{R206H}. Immunoblotting corroborated these findings at the protein level: while ACVR1^{WT} levels remained stable (Figure 2H), ACVR1^{R206H} protein abundance was significantly reduced (Figure 2I), with mMOE3 achieving the strongest knockdown (87.74 ± 1.86%). These results demonstrate that a single-base 2′OMe modification improves allele-selective silencing at both the RNA and protein levels.

We next investigated whether the modified gapmers could mitigate osteogenic differentiation driven by *ACVR1*^{R206H} activity. In FOP, the R206H mutation renders the receptor aberrantly responsive to activin A,[4,46] which induces osteogenic markers, e.g., alkaline phosphatase (ALP), in mesenchymal cells derived from FOP iPSCs.[46–48] C2C12 cells expressing V5-tagged *ACVR1*^{R206H} were treated with recombinant human activin A (rhActivin A) and analyzed for ALP expression and activity. ALP staining revealed robust induction in *ACVR1*^{R206H}-expressing cells, which was significantly reduced by gapmer treatment, with mLNA16 and mMOE3 showing superior inhibition compared to unmodified gapmers (Figures 2J and 2K). To assess later stages of osteogenic differentiation, we cultured *ACVR1*^{R206H}-transfected C2C12 cells in osteogenic medium and examined calcium deposition via Alizarin Red S (ARS) staining after 21 days. Cells expressing mutant *ACVR1* showed intense ARS staining, which was markedly reduced following gapmer treatment. Importantly, both 2′OMe-modified gapmers significantly outperformed their unmodified counterparts in suppressing mineralization (Figures 2L and 2M). RT-qPCR analyses of *Alpl* and *Runx2* expression further supported these observations, with mLNA16 and mMOE3 showing the strongest suppression of osteogenic gene expression 48 h post-treatment (Figure S3).

These findings demonstrate that incorporation of a single 2′OMe modification at gap position 2 enhances allele selectivity of antisense gapmers and attenuates *ACVR1*^{R206H}-driven osteogenic differentiation in an engineered C2C12 model, supporting further evaluation of this strategy for therapeutic development in FOP.

Single-base 2′OMe modification improves cell-level safety profiles in FOP patient-derived fibroblasts

To evaluate the cytocompatibility of gapmer treatments, cytotoxicity, cell vitality, and apoptotic activity were quantified in FOP patient-derived fibroblasts using the ApoTox-Glo triplex assay, which

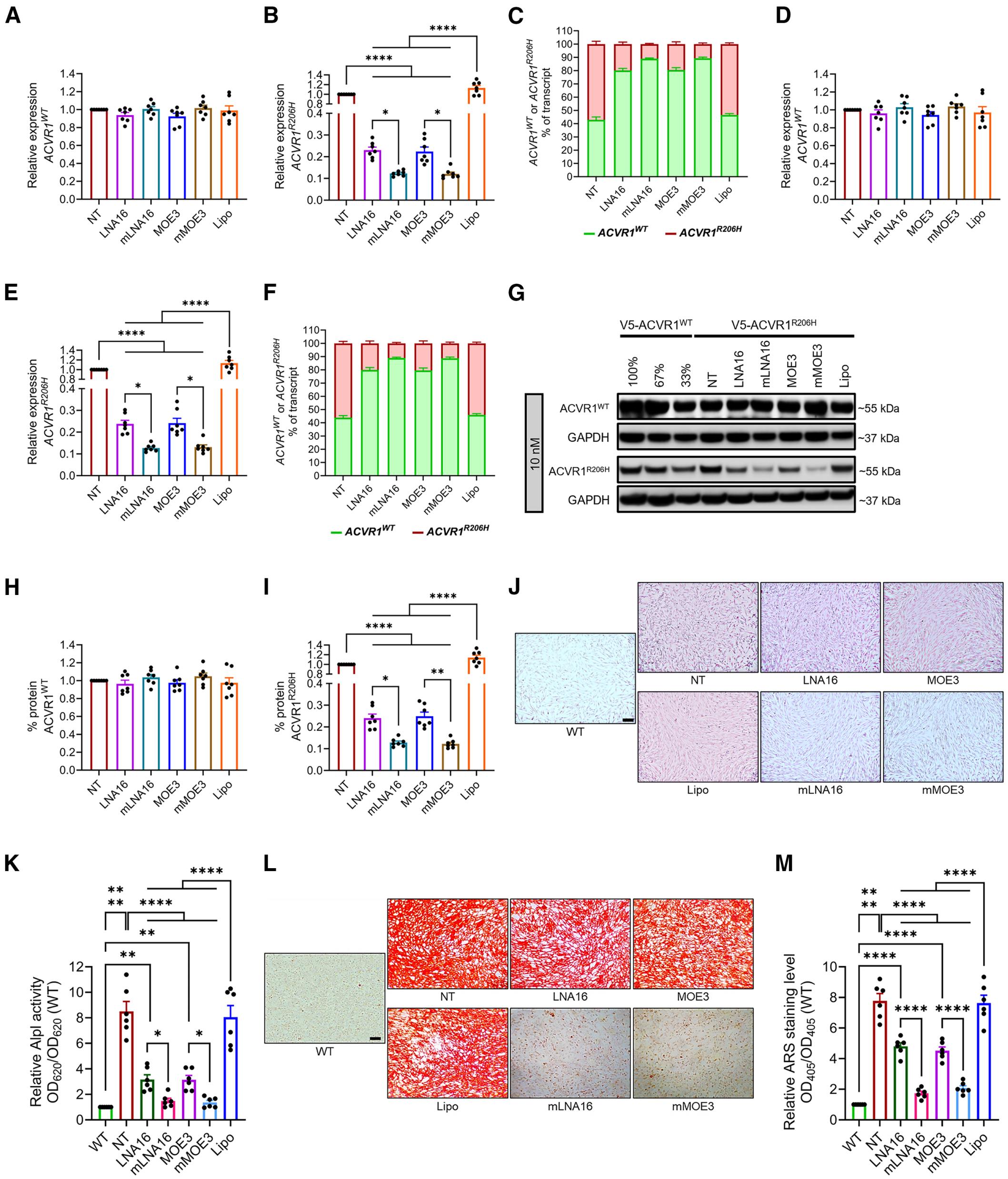

Figure 2. Single-base 2′OMe modification enhances allele selectivity and suppresses *ACVR1*^{R206H}-induced osteogenic differentiation *in vitro*

(A and B) RT-qPCR quantification of *ACVR1*^{WT} (A) and *ACVR1*^{R206H} (B) transcripts in FOP patient-derived fibroblasts treated with 10 nM LNA16 or MOE3 gapmers, with or without a single 2′OMe modification at the second position of the gap region. (C) Allelic fractions of *ACVR1*^{WT} and *ACVR1*^{R206H} transcripts post-treatment as calculated from total *ACVR1* expression. Green and red bars represent *ACVR1*^{WT} and *ACVR1*^{R206H} transcript fractions, respectively. (D and E) RT-qPCR quantification of *ACVR1*^{WT} (D) and *ACVR1*^{R206H} (E) transcripts in C2C12 myoblasts transfected with V5-tagged *ACVR1*^{WT} or *ACVR1*^{R206H} constructs and treated with 10 nM gapmers for 48 h. (F) Allelic fraction analysis of *ACVR1*^{WT} and *ACVR1*^{R206H} transcripts in cells from (D and E), similarly as (C). (G) Representative immunoblot showing V5-tagged *ACVR1*^{WT} and *ACVR1*^{R206H} protein levels in transfected C2C12 cells after 10 nM gapmer treatment. GAPDH served as a loading control. (H and I) Densitometric quantification of *ACVR1*^{WT} (H) and ACVR1^{R206H} (I) protein expression from (G), normalized to GAPDH and expressed relative to the non-treated control. (J) Representative images of alkaline phosphatase (ALP) staining in C2C12 cells expressing V5-*ACVR1*^{R206H} or *ACVR1*^{WT}, treated with 10 nM gapmers and stimulated with recombinant human activin A (100 ng/mL) for 48 h. (K) Quantification of secreted ALP enzymatic activity in conditioned media collected from cells in (J), normalized to the *ACVR1*^{WT} media. (L) Representative images of ARS staining performed 21 days after osteogenic differentiation induction in C2C12 cells transfected with V5-*ACVR1*^{R206H} or *ACVR1*^{WT} and treated with gapmers. (M) Quantification of ARS staining from (L), normalized to the *ACVR1*^{WT} control. Statistics, one-way ANOVA with Tukey's multiple comparisons test; $^{*}p < 0.05$, $^{**}p < 0.01$, $^{****}p < 0.0001$. Scale bars, 250 μm. Data are represented as mean ± standard error of the mean (n = 6–7). NT, non-treated; Lipo, lipofectamine 3000 (a commercially available transfection reagent).

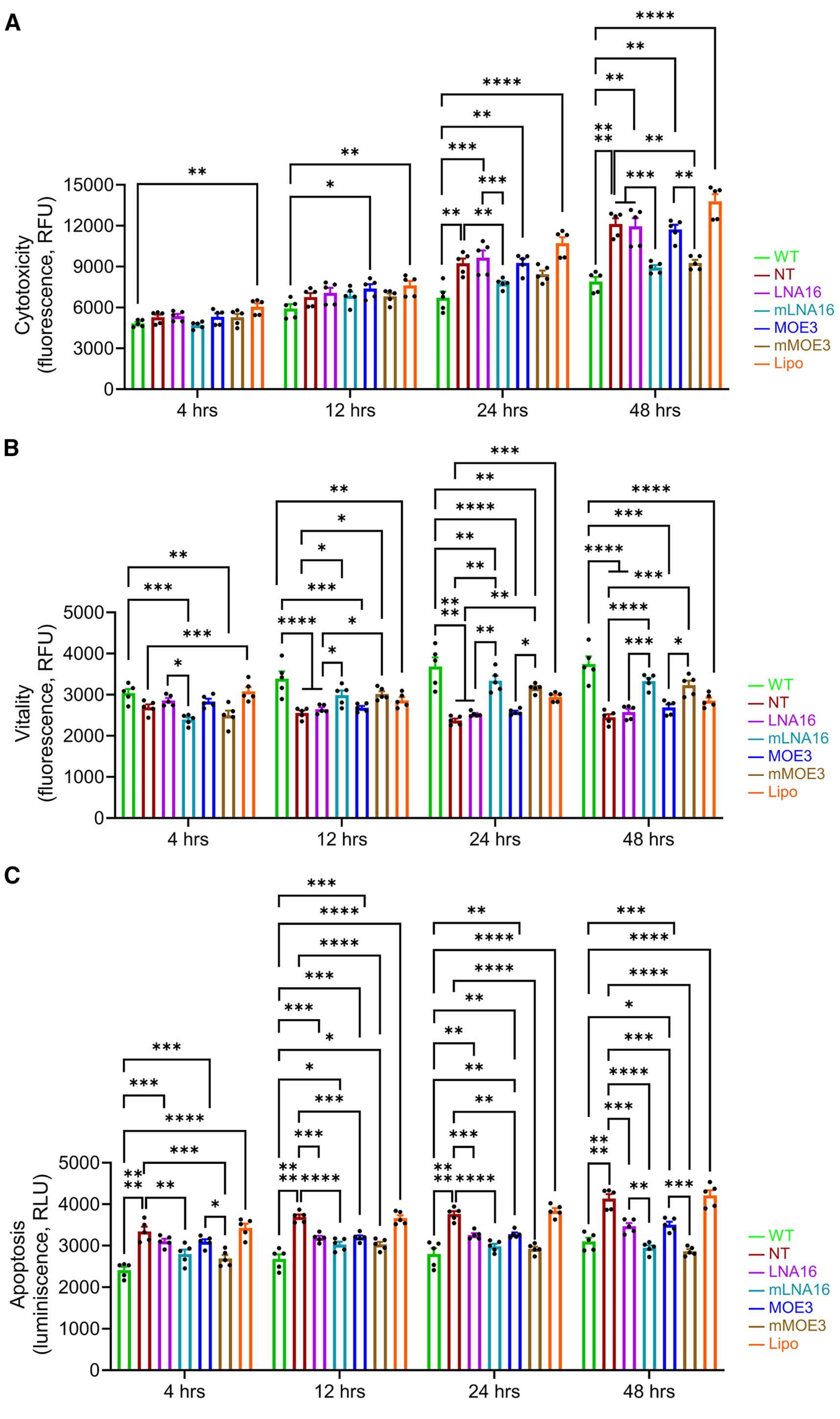

Figure 3. Single-base 2′OMe modification mitigates cytotoxicity and apoptosis while preserving cell vitality following gapmer treatment in FOP fibroblasts

(A) Cytotoxicity assessment using the ApoTox-Glo triplex assay. Healthy and FOP patient-derived fibroblasts treated with 10 nM gapmers (with or without a single 2′OMe modification) were assessed at 4, 12, 24, and 48 h post-treatment. Cytotoxicity is presented as relative fluorescence units (RFU), with higher values indicating greater cytotoxicity. (B) Cell vitality assessment from the same assay conditions. Vitality is presented as RFU, with higher values indicating increased cell viability. (C) Apoptosis assessment measured by caspase-3/7 activity, presented as relative luminescence units (RLU), with higher values indicating increased apoptosis. Statistics, one-way ANOVA with Tukey's multiple comparisons test; *$p < 0.05$, **$p < 0.01$, ****$p < 0.0001$. Data are represented as mean ± standard error of the mean ($n = 5$). NT, non-treated; Lipo, lipofectamine 3000 (a commercially available transfection reagent).

simultaneously measures loss of membrane integrity (cytotoxicity), intracellular protease activity (cell vitality/metabolic activity), and caspase-3/7 activation (apoptosis) within the same sample.

Treatment with gapmers at 10 nM induced increased cytotoxicity at 24 and 48 h relative to untreated controls, as indicated by elevated fluorescence intensity (Figure 3A). Incorporation of a single 2′OMe modification significantly reduced cytotoxicity compared to unmodified gapmers across both time points. Parallel assessment of cell vitality revealed a time-dependent decline in untreated and unmodified gapmer-treated cells, whereas 2′OMe-modified gapmers preserved higher vitality signals at 24 and 48 h post-treatment (Figure 3B). Apoptotic activity, measured by luminescence from caspase-3/7 substrates, was elevated relative to healthy controls at all assessed time points (Figure 3C). Gapmer treatment reduced caspase-3/7 activity relative to non-treated cells; notably, 2′OMe-modified gapmers achieved greater reductions in apoptotic signaling compared to their unmodified counterparts, suggesting improved tolerability at equivalent doses.

Beyond these cytocompatibility indices, we also evaluated the *in vitro* off-target effects of the gapmer treatments. To assess the off-target profiles of LNA16 and MOE3, we compiled a list of potential target sequences containing up to one (LNA) or two (MOE) base mismatches (Table S1). At a 10 nM dose, these predicted off-target genes did not exhibit severe or widespread targeted knockdown, though a minor reduction in *EXTL3* was observed with LNA16 (Figure S4). In addition, we looked into the expression of *P54nrb* and *PSF*, two genes known to be broadly affected by ASO treatments and found that neither was impacted significantly by LNA16 or MOE3 (Figure S4).[43,49,50]

These results suggest that the introduction of a single 2′OMe base may improve acute tolerability in FOP fibroblasts by reducing cytotoxicity and caspase-3/7 activation while maintaining cellular metabolic activity, with no evidence of major off-target effects in the panel of potential off-target genes assessed, supporting its potential utility for improving the cytocompatibility of antisense therapeutics targeting $ACVR1^{R206H}$.

Single-base 2′OMe modification improves skeletal muscle bioavailability and preserves safety profiles *in vivo*

To gain insights into the *in vivo* performance of 2′OMe-modified gapmers, we quantified their biodistribution and evaluated systemic safety following systemic administration. Wild-type B6 mice received a single retro-orbital injection of 11 mg/kg of either unmodified or 2′OMe-modified gapmers, and tissues were collected 72 h post-injection.

Tissue gapmer concentrations were quantified using a non-competitive hybridization-based ELISA (Figures 4A–4J). Both unmodified and 2′OMe-modified gapmers predominantly accumulated in the kidney and liver (Figures 4E and 4F). However, 2′OMe-modified gapmers demonstrated significantly reduced accumulation in these clearance organs relative to unmodified versions. Importantly, the 2′OMe-modified gapmers showed enhanced uptake in skeletal muscles, including quadriceps, tibialis anterior, and diaphragm, as well as the Achilles tendon (Figures 4A–4C, 4H, and 4J). Brain uptake remained negligible across all conditions, and heart and spleen uptake were unchanged (Figures 4B–4D and 4I). These findings suggest that a single 2′OMe modification does not abolish the predominant liver/kidney uptake of gapmers but is associated with relatively higher skeletal muscle and tendon levels at 72 h post-injection in this acute wild-type mouse study.

To characterize systemic tolerability, serum biochemistry was evaluated 72 h post-treatment (Figures 4K–4T). Elevations in hepatic enzymes, e.g., ALP, alanine transaminase (ALT), and aspartate aminotransferase (AST), were observed following administration of unmodified gapmers, with consistently lower levels detected in 2′OMe-modified gapmer-treated mice (Figures 4K–4M). Renal function markers, e.g., blood urea nitrogen (BUN), creatinine, exhibited mild elevations with unmodified gapmers, particularly the LNA-based construct, while 2′OMe-modified gapmers preserved near-baseline levels (Figures 4N and 4P). Total protein (T.Prot) and albumin levels were elevated following gapmer treatment irrespective of modification, although albumin-to-globulin ratios were significantly less perturbed by 2′OMe modification (Figures 4R–4T). Serum creatine kinase (CPK) and total bilirubin (T.Bil) remained unchanged across groups (Figures 4O and 4Q). Also, given the sensitivity of renal stress to gapmer exposure, urinary kidney injury molecule 1 (KIM1) levels were measured as an early biomarker of nephrotoxicity. KIM1 levels were significantly elevated in mice treated with unmodified gapmers but remained unchanged in mice receiving 2′OMe-modified gapmers (Figure 4U).

Histological analyses of liver and kidney tissues 3 days post-treatment revealed that treatment with unmodified gapmers resulted in mild morphological alterations, including hepatic cytoplasmic pallor and minor renal tubular dilation with minimal vacuolization (Figure S5). The 2′OMe-modified gapmers, on the other hand, visibly mitigated these acute architectural perturbations. No overt signs of necrotic lesions, severe inflammatory infiltrates, or early fibrotic changes were observed in any of the groups. Molecular assessments of tissue stress responses by RT-qPCR showed upregulation of tubular stress (*Havcr1* and *Lcn2*), oxidative stress (*Hmox1*), and pro-fibrotic (*Tgfb1*) and inflammatory markers (*Tnf*) in the liver and kidney following unmodified gapmer treatment, with substantially lower induction following 2′OMe-modified gapmer administration (Figure S6). Renal stress signatures were particularly pronounced with unmodified LNA-based gapmers and markedly attenuated with 2′OMe modification. However, by 15-days post-treatment, both histological architecture and the transcriptional expression of all evaluated stress markers had normalized to baseline levels across the board (Figures S5 and S6). These results suggest that although unmodified gapmers induce mild hepatic and renal stress, these changes are transient, non-progressive, and reversible, as evidenced by the normalization of tissue stress markers and the absence of pathological lesions in histological analysis.

Taken together with the biodistribution studies, these results indicate that incorporation of a single 2′OMe base significantly improves skeletal muscle and tendon bioavailability while reducing early hepatic and renal stress markers in this acute single-dose wild-type mouse study.

Overall, our findings indicate that single-base 2′OMe modification improves the allele-selectivity of gapmers targeting $ACVR1^{R206H}$ in our *in vitro* models and is associated with shifts in apparent tissue biodistribution and short-term safety readouts *in vitro* and *in vivo*. These data provide rationale for further evaluation of 2′OMe-modified gapmers in disease-relevant FOP models to establish therapeutic feasibility of this strategy *in vivo*.

DISCUSSION

FOP remains one of the most devastating and therapeutically intractable genetic disorders, primarily driven by a single point mutation in *ACVR1* that disrupts key processes of tissue homeostasis.[4,7,8] Despite significant advancements in understanding its molecular mechanisms, effective therapeutic options for FOP remain scarce, primarily due to the challenges of selectively targeting the pathogenic *ACVR1* allele while preserving the essential functions of the wild-type receptor.[4,51] In this study, we developed and rigorously evaluated allele-specific antisense gapmers incorporating a single 2′OMe modification to enhance therapeutic precision and safety. Our findings suggest that rational chemical optimization of antisense gapmers can improve allele selectivity, reduce cytotoxicity in our *in vitro* assays, and modulate acute tissue distribution, all of which are important considerations for advancing FOP antisense strategies in preclinical development.

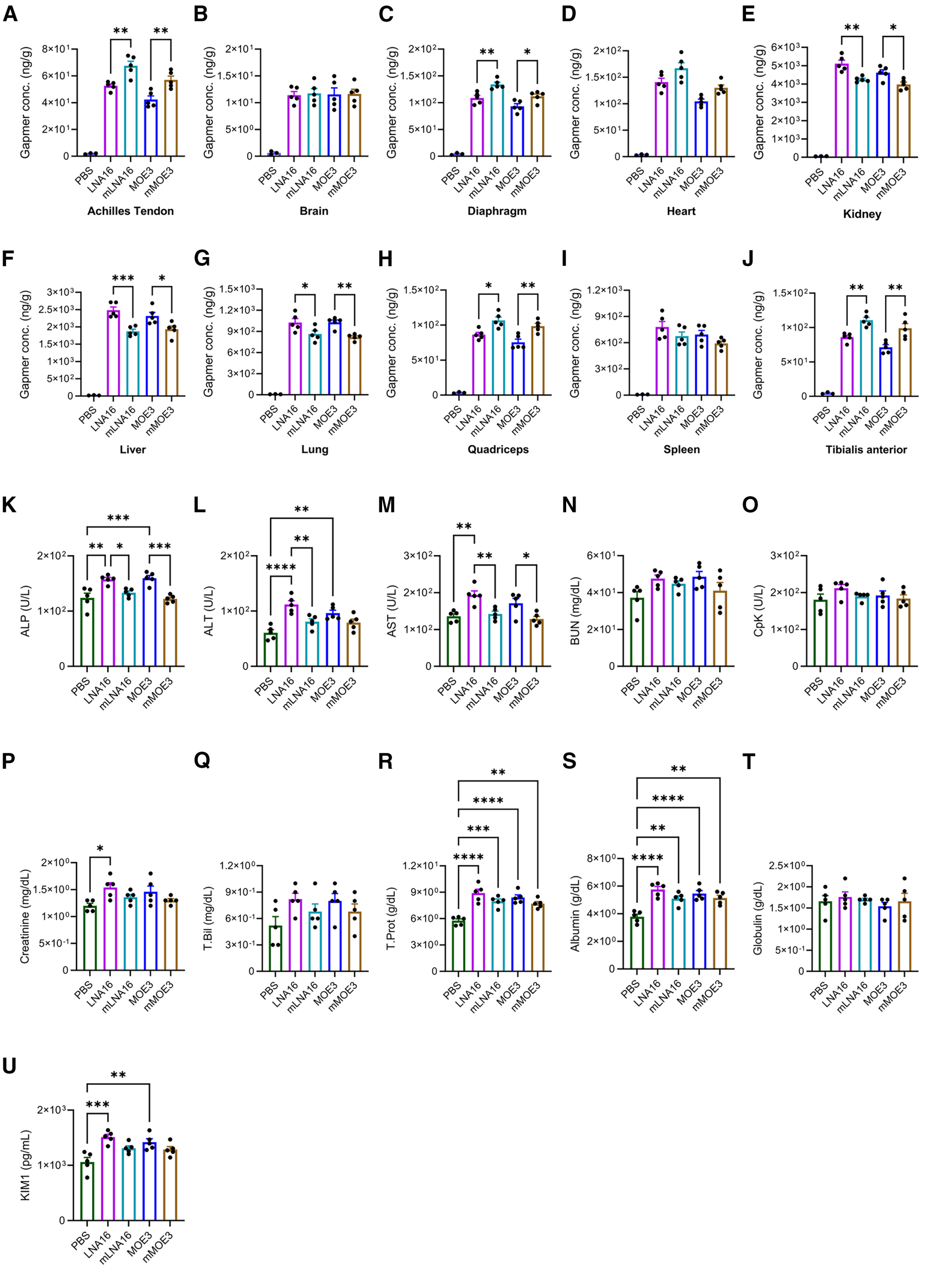

Figure 4. Single-base 2′OMe modification improves skeletal muscle bioavailability and mitigates hepatic and renal stress markers following systemic gapmer delivery

(A–J) Gapmer concentrations measured in different tissue samples, e.g., (A) Achilles tendon, (B) brain, (C) diaphragm, (D) heart, (E) kidney, (F) liver, (G) lung, (H) quadriceps, (I) spleen, and (J) tibialis anterior (TA) measured by a non-competitive hybridization-based ELISA. (K–T) Serum biochemistry analysis assessing hepatic and renal markers: ALP (K), ALT (L), AST (M), BUN (N), CPK (O), creatinine (P), T.Bil (Q), T.Prot (R), albumin (S), and globulin (T). (U) Kidney injury molecule 1 (KIM1) levels in urine samples as a marker of renal tubular stress. Across the board, gapmers (11 mg/kg) were administered retro-orbitally in wild-type B6 mice. Tissue and blood samples were collected 72 h post-injection. Statistics, one-way ANOVA with Tukey's multiple comparisons test; *$p < 0.05$, **$p < 0.01$, ***$p < 0.001$, ****$p < 0.0001$. Data are represented as mean ± standard error of the mean ($n = 5$).

Our work builds on previous efforts to exploit antisense platforms for mutant allele targeting but introduces several key innovations.[19,24] First, we designed and validated a panel of LNA- and MOE-based gapmers targeting the *ACVR1*^{R206H} allele and demonstrated that incorporating a T→G mismatch improves mutant allele discrimination without sacrificing therapeutic potency. Second, we introduced a single 2′OMe modification at the second position of the gap region, a site previously implicated in modulating protein-binding and toxicity, and observed that this strategic alteration preserved mutant allele knockdown while improving cytocompatibility in FOP patient-derived immortalized fibroblasts and attenuating acute hepatic and renal readouts *in vivo*.[44,45] Our 2′OMe-modified gapmers retained high mutant allele knockdown efficacy in both patient-derived fibroblasts and engineered myoblast models while substantially reducing cytotoxicity, apoptotic signaling, and off-target effects on the wild-type *ACVR1* allele. These results not only validate the utility of single-base chemical modifications for antisense therapeutics but also demonstrate the flexibility of gapmer design for allele-specific applications in dominant genetic disorders like FOP. Mechanistically, we hypothesize that the enhanced allele selectivity observed with single-base 2′OMe-modified gapmers arises from a compounded destabilization effect. Since the gapmers are designed to perfectly match the mutant *ACVR1*^{R206H} allele, they inherently introduce a mismatch when binding to the wild-type transcript. The addition of a 2′OMe sugar at a central gap position acts as a structural perturbation that impairs RNase H1 recruitment. The wild-type duplex, burdened by both a sequence mismatch and the chemical interference, may fall below the thermodynamic threshold required for efficient RNase H1 cleavage. On the other hand, the perfectly matched mutant duplex maintains sufficient stability to remain a viable substrate. We could not directly measure RNase H cleavage or the stability of modified gapmer/RNA duplexes compared to the unmodified ones, and this hypothesis is solely based on previous research on sugar modifications and their effect on RNase H recruitment, which showed that sugar modifications at key positions in gapmers can influence RNase H recruitment and cleavage in a mismatch-sensitive manner.[52,53]

In vivo, our data reveal that the 2′OMe modification is associated with altered tissue distribution of gapmers, favoring skeletal muscle and tendon tissues while reducing accumulation in clearance organs, e.g., the kidney and liver. This shift in tissue biodistribution is particularly significant given the muscle-predominant pathophysiology of FOP and underscores the need for therapeutic exposure in affected tissues. One possible explanation for these distribution differences, based on prior work with 2′OMe-modified PS-modified ASOs, is altered protein-binding behavior, which can influence renal filtration, hepatic uptake, and circulation time.[44,45] We did not directly assess plasma pharmacokinetics or protein binding in this study, so these mechanisms remain speculative and will require targeted follow-up experiments. Therefore, future pharmacokinetic and protein-binding studies will be important to determine whether these differences reflect altered clearance, distribution kinetics, or both. In parallel, although unmodified gapmers induced mild elevations in hepatic and renal stress markers, these changes were transient, non-progressive, and reversible, as evidenced by preserved tissue architecture, absence of pathological lesions, and normalization of molecular stress signatures following 2′OMe modification. These findings underscore the critical role of minor chemical adjustments in enhancing the tolerability of gapmers without compromising their efficacy, a balance that remains a major challenge in the clinical development of antisense therapeutics.[26,44,45]

While our study demonstrates a significant advancement in allele-selective silencing, it is essential to compare our findings within the broader landscape of FOP therapies. Currently, the only FDA-approved treatment for FOP is palovarotene, a retinoic acid receptor-γ agonist that inhibits HO.[54] However, it has notable side effects, including hepatotoxicity, and its efficacy in pediatric patients is not fully optimized.[55–57] In contrast, our 2′OMe-modified gapmers selectively reduced the pathogenic *ACVR1*^{R206H} allele while largely sparing the wild-type allele in our cell-based assays and improved cytocompatibility readouts relative to unmodified gapmers, suggesting a lower likelihood of undesired wild-type allele suppression and improved cytocompatibility in the tested models. This strategy conceptually differs from palovarotene, which broadly modulates retinoic acid signaling; however, formal safety and efficacy comparisons between antisense approaches and palovarotene have not yet been performed.[4,51]

In addition to palovarotene, several other experimental therapies for FOP are under investigation.[4,58] Small molecules like Zilurgisertib (INCB000928), a selective ALK2 inhibitor, target aberrant activin A signaling, but clinical trials have raised concerns over treatment-emergent adverse events (TEAEs), e.g., thrombocytopenia, epistaxis, asthenia, tachycardia, and hypersensitivity.[59] Similarly, AAV-based gene therapies, e.g., the strategy proposed by Yang et al. (2022), combine codon-optimized *ACVR1* gene addition with *ACVR1*^{R206H}-specific silencing using synthetic miRNAs.[15] While promising, AAV-based approaches face challenges like limited tissue targeting, packaging constraints, and immunogenicity, especially in FOP patients.[4,60] In comparison, our gapmer strategy bypasses these viral delivery complications and offers a direct, allele-specific silencing approach that may reduce certain vector-related immunogenic risks relative to AAV-based gene delivery[61–64]; however, dedicated immunogenicity studies will be required to define the innate and adaptive immune responses to these gapmers in FOP-relevant settings. Gapmers can be integrated with other therapies, creating opportunities for combination treatments.[4,51]

Several antibody-based therapies, including Garetosmab[65,66] and Andecaliximab,[67] target activin A and block activin receptor signaling, respectively. Other promising candidates include Saracatinib,[14] which targets Src-family kinases involved in FOP-related signaling, and Fidrisertib (IPN60130/BLU-782),[68] an ALK2 inhibitor. While these therapies have demonstrated some success in preclinical trials, their broad activity can lead to undesirable effects on normal bone metabolism and other tissues. For instance, blocking

activin A signaling can have unintended consequences on other BMP-related pathways that are crucial for normal development and function.[13] Our gapmer-based strategy specifically targets the mutant *ACVR1* allele, thereby offering a more localized and precise therapeutic effect, with fewer risks of interfering with other critical signaling pathways.

Similarly, siRNA-based approaches show promise in silencing *ACVR1*^{R206H}, but they face challenges related to transient knockdown, off-target effects, and delivery issues.[19] In contrast, gapmer approaches act through RNase H-mediated degradation of the target transcript, which can provide sustained knockdown with reduced chances of undesired immune activation and enhanced specificity. In our prior works, we reported that allele-selective LNA gapmers result in a significantly stronger knockdown of *ACVR1*^{R206H} in comparison to siRNAs *in vitro*. In the current study, we did not directly compare durability or immunogenicity between gapmers and siRNA candidates and any differences in these properties remain to be established experimentally. In addition, exon-skipping strategies have shown potential in FOP,[69] similar to their success in muscular dystrophies. However, this approach faces challenges related to mutant allele-selective exon targeting and efficient delivery. Unlike exon-skipping, which aims to bypass mutations in a non-allele-selective manner, our gapmer strategy precisely targets the mutation, providing a more targeted and potentially more effective solution for FOP. In general, while all these experimental therapies for FOP show promise, our 2′OMe-modified gapmer strategy offers conceptual advantages in allele selectivity and the potential for improved safety, although direct comparative studies with other modalities are still lacking. By selectively reducing the mutant *ACVR1*^{R206H} allele in our models while improving cytocompatibility and acute stress readouts relative to unmodified gapmers, this approach represents a promising complementary modality that demands further comparative evaluation.

One important aspect of our approach is that it presents a unique opportunity for synergistic combination therapies.[4,16,51,70] A promising approach would be the silence-and-replace strategy, where gapmer-mediated depletion of the toxic *ACVR1*^{R206H} transcript is paired with a gene-addition approach, e.g., AAV-mediated delivery of codon-optimized *ACVR1*^{WT} to restore healthy signaling. Besides, integrating the gapmer approach with small molecule approaches, e.g., palovarotene or ALK2 inhibitors, could facilitate significant dose-sparing. This combination strategy could enhance the therapeutic window, improving HO blockade while mitigating severe, dose-dependent adverse effects, often associated with current pharmacological standards. Moreover, the pharmacokinetic profile of our designed gapmers aligns with the clinical realities of FOP management. Given the progressive nature of the disease and the risks of continuous systemic target engagement, the most realistic application for gapmer therapy is perhaps episodic administration following soft tissue trauma or the onset of a flare-up. Since gapmers typically exhibit prolonged intracellular half-lives, often spanning several weeks,[71–77] a single acute dose or short multi-dose regimen during a flare-up could provide sustained local suppression of pathogenic signaling precisely during the critical window of HO.

Despite the promise of our findings, several limitations must be addressed. First, we evaluated acute safety and biodistribution only at 3 days post-injection following a single systemic dose in wild-type mice. The high species specificity of our lead candidates precluded efficacy testing in traditional murine FOP models. Longer-term and repeat-dose studies in disease-relevant, humanized knock-in models are essential to fully characterize chronic toxicity, immunogenicity, pharmacodynamics, and therapeutic efficacy. Second, although our data demonstrate effective mutant allele silencing and inhibition of osteogenic markers *in vitro*, the impact of 2′OMe-modified gapmers on HO in a suitable *in vivo* model has yet to be assessed. Accordingly, these data should be interpreted cautiously. Third, we did not include a scrambled or random-sequence gapmer control, thus we cannot fully exclude sequence-independent effects of phosphorothioated oligonucleotides or transfection. Consequently, we interpret phenotypic changes as likely due to the knockdown of *ACVR1*^{R206H} but cannot attribute them exclusively to on-target effects. Moreover, our mechanistic studies relied on FOP patient-derived immortalized fibroblasts and *ACVR1*^{R206H}-transfected C2C12 cells under osteogenic induction, which represent only surrogate models of FOP pathology. Thus, the functional osteogenic readouts we report are limited to engineered myoblast models rather than bona fide FOP progenitor cells. To note, we did not observe robust ARS mineralization responses in FOP fibroblasts under our conditions. Additionally, while our biodistribution and safety assessments were conducted in wild-type mice rather than FOP-specific models, future studies in relevant FOP mouse models will be crucial for determining disease-modifying effects and ensuring the clinical relevance of our findings. Furthermore, while the 2′OMe modification significantly improves cytocompatibility, further investigation into potential off-target RNA cleavage events at transcriptome-wide resolution is warranted to comprehensively assess the safety profile. Also, since we did not use any external cytotoxic positive control in these assays, we interpreted these data as comparative effects across gapmer chemistries and, as such, they may not be indicative of absolute levels of toxicity. Lastly, regarding the statistical analyses used in the study, they were primarily exploratory, therefore, we emphasize the consistency and size of effect sizes rather than focusing on isolated statistical significance values. Collectively, our findings should be viewed as a preliminary single-dose, acute-time-point proof-of-concept study that will require replication, extension, and validation in suitable models and dosing paradigms.

Despite these limitations, this study provides a foundational proof-of-concept that single-base chemical modifications can modulate, and may improve, the therapeutic window of allele-specific gapmers. Our results complement and extend prior findings on the pharmacological behavior of PS-modified ASOs and support the growing recognition that selective chemical editing of ASO backbones offers a powerful means to optimize safety profiles.[26] Comparatively, while earlier reports have demonstrated the feasibility of allele-selective targeting in FOP using siRNA or gapmer approaches, our work uniquely highlights the synergistic benefit of combining

mismatch-based design with minimal 2′OMe substitution to achieve high allele specificity alongside improved *in vivo* bioavailability and safety.[19,24,26,44,45]

Future studies should focus on evaluating the long-term therapeutic efficacy of the 2′OMe-modified LNA and MOE gapmers in suitable humanized preclinical FOP models. This includes assessing their ability to prevent (and potentially reverse) HO *in vivo*. These should include direct, head-to-head comparative cohorts with existing clinical standards, e.g., palovarotene, to rigorously benchmark the therapeutic window, efficacy, and skeletal safety profile of this allele-specific gene silencing approach. This could potentially bridge this gap between pharmacokinetic safety and disease-modifying efficacy. Furthermore, comparative studies with existing treatment modalities, e.g., palovarotene and other *ACVR1*-targeted therapies, would be invaluable for contextualizing the potential advantages of allele-specific gene silencing approaches. Additionally, our findings align with established models of RNase H1-mediated cleavage modulation by sugar modifications; however, direct evaluations through a set of comprehensive RNase H1 cleavage kinetics assays and molecular dynamics simulations remain a critical next step to resolve the precise structural basis of this enhanced selectivity. Understanding the precise biophysical parameters by which a single 2′OMe modification at the second position within the central DNA region modulates mismatch discrimination would provide invaluable insights into rational gapmer design for allele-selective applications and could further guide future chemical optimization strategies to enhance precision, efficacy, and safety. Also, exploring alternative chemical modifications, e.g., cEt, or newer hybrid designs may provide further improvements in efficacy, durability, and safety.[26] In order to overcome the inherent delivery barriers to skeletal muscle and fascial tissues, future iterations of these gapmers would benefit significantly from the addition of extra-hepatic targeting ligands.[26,78] Bioconjugation platforms, e.g., transferrin receptor 1 (TfR1)-targeting antibodies, cell-penetrating peptides (CPPs), or nanoparticle delivery shuttles could help drive the gapmer into pathogenic fibro/adipogenic progenitors.[79–82] In addition, scaling up the production of ASO drugs in a cost-effective manner remains an important consideration for their broader clinical adoption. However, 2′OMe bases have been in production in laboratory conditions since the late 60s and have been in commercial production for decades.[83] From a translational and manufacturing perspective, the incorporation of a single 2′OMe substitution relies on standard solid-phase phosphoramidite chemistry. This modification does not compromise synthesis yield or purity, ensuring that clinical scale-up remains highly feasible and cost-effective compared to more complex structural modifications. Ultimately, the translational advancement of this platform will depend on demonstrating robust, durable therapeutic benefits in disease-relevant models while maintaining a high margin of safety.

Overall, our findings suggest that single-base 2′OMe modification is a promising strategy to optimize allele-selective antisense gapmers targeting $ACVR1^{R206H}$. This work provides an initial experimental basis for further development of antisense therapeutics for FOP and indicates the broader potential of precision chemical tuning to refine the safety and distribution profiles of next-generation genetic therapies for dominant genetic disorders.

MATERIALS AND METHODS

Ethics statement

All experiments involving human and animal samples were approved by the Research Ethics Office (REO) at the University of Alberta. The use of immortalized human fibroblast cell lines was reviewed and approved by the Human Research Ethics Boards, REO (Pro00079871), while animal studies received authorization from the Animal Care and Use Committees, REO (AUP00000365).

Gapmer design, synthesis, transfection, and off-target effect evaluation

All gapmers used in this study were fully PS-modified to improve nuclease resistance and synthesized commercially by Exiqon and/or Integrated DNA Technologies (IDT). LNA gapmers were designed with 16 nucleotides, including LNA-modified regions at the first and last three nucleotides. MOE gapmers were 20 nucleotides long, with 5-nucleotide MOE-modified regions flanking a 10-nucleotide DNA core. All gapmers used in this study were designed to target *ACVR1* transcripts (Figure 1A).

Potential off-target sequences were identified at the GGGenome web interface (https://gggenome.dbcls.jp/), a tool specifically optimized for searching databases with short sequence inputs compared to standard BLAST (Table S1).[84] The RefSeq human RNA database (release 230; May 2025) was queried for sequences complementary to LNA16r, LNA16s, MOE3r, and MOE3s, allowing for a maximum of one mismatch for the LNA sequences and two mismatches for the MOE sequences. The top hits against LNA16 (i.e., LNA16s) and MOE3 (i.e., MOE3r) were selected for further evaluation using RT-qPCR.

Cell culture

Immortalized human fibroblasts were obtained from the Coriell Cell Repository (NJ, USA). Two cell lines were used: GM00513 (referred to as FOP patient-derived fibroblasts) and GM23815 (referred to as healthy fibroblasts). GM00513 is an immortalized fibroblast line derived from a skin biopsy of a 16-year-old female patient with genetically confirmed FOP. GM23815 is an immortalized fibroblast line derived from a skin biopsy of a healthy 22-year-old male individual. Both cell lines were maintained in Dulbecco's modified Eagle medium/nutrient mixture F-12 (DMEM/F-12) medium supplemented with 10% fetal bovine serum (FBS; Sigma-Aldrich, St. Louis, MO, USA), 1% L-glutamine (Gibco, Grand Island, NY, USA), and 0.5% penicillin-streptomycin (Gibco).

Murine C2C12 myoblasts (American Type Culture Collection, VA, USA) were cultured in DMEM/F-12 medium supplemented with 15% FBS and 0.5% penicillin-streptomycin under standard conditions.

Transfection

FOP patient-derived and healthy fibroblasts were seeded onto 12 or 24 well plates (BioLite/Thermo Scientific) at a density of 50,000 cells/ cm^2 (Figure 1B). Gapmers were transfected using Lipofectamine 3000 (Thermo Fisher Scientific) in Opti-MEM reduced serum medium supplemented with GlutaMAX (Thermo Fisher Scientific), following the manufacturer's protocol. The transfection mixture was diluted 1:5 in complete growth medium before being added to the cells. Fibroblasts treated with transfection reagents without gapmers served as non-treated controls, and healthy fibroblasts cultured under identical conditions served as healthy controls. The final concentrations of gapmers in the media, i.e., 10 and 100 nM, were selected to represent the anticipated therapeutic range and a relatively high-exposure stress-test condition, respectively, based on prior potency benchmarks.

For C2C12 myoblasts, cells were seeded at 50,000 cells/cm^2 and cultured for 24 h before transfection. V5-tagged $ACVR1^{WT}$ or $ACVR1^{R206H}$ plasmids were cotransfected with gapmers using Lipofectamine 3000. The constructs were driven by an EF-1α promoter and contained a C-terminal V5 epitope tag. Cells were either harvested 48 h post-transfection or maintained under osteogenic conditions until further analysis for alkaline phosphatase or ARS staining.

RNA extraction, cDNA synthesis, and RT-qPCR

Total RNA was extracted from cultured cells using the RNeasy Mini Kit (QIAGEN, Germany) according to the manufacturer's instructions. To remove residual genomic DNA completely, lysates were subjected to on-column DNase digestion using an RNase-Free DNase kit (QIAGEN), as described previously.[85] For frozen tissue samples, RNA was isolated from 20 to 30 sections (20 μm thick) using TRIzol reagent (Thermo Fisher Scientific), with a modification of overnight incubation at −80°C prior to homogenization to maximize RNA yield.

For cDNA synthesis, 1,400 ng of total RNA was reverse transcribed using the SuperScript IV One-Step RT-PCR System (Invitrogen, Vilnius, Lithuania) with 0.5 μg of Oligo(dT)$_{12-18}$ primers (Invitrogen, Carlsbad, CA) in a 20 μL reaction volume. A no-template control containing nuclease-free water was included in each batch. The resulting cDNA was used as a template for RT-qPCR on a QuantStudio 3 Real-Time PCR System (Applied Biosystems, Carlsbad, CA, USA).

TaqMan gene expression assays (Thermo Fisher Scientific) were used to quantify total *ACVR1* (Hs00153836_m1), *Alpl* (Mm00475834_m1), *Runx2* (Mm00501584_m1), and *RPS18*/*rps18* (Hs01375212_g1/Mm02601777_g1) transcripts. Allele-specific quantification of *ACVR1* was performed using a custom TaqMan SNP genotyping assay (Assay ID ANKA3PJ; Thermo Fisher Scientific) with the following primers: forward, CTCTGGTCTTCCTTTTCTGGTACAA; reverse, CCCGACACACTCCAACAGT. Reporter probes were VIC-labeled AGTGGCTCGCCAGATT (wild type) and FAM-labeled CAGTGGC TCACCAGATT (mutant). Reactions were prepared with TaqMan Fast Advanced Master Mix (Thermo Fisher Scientific) according to the manufacturer's instructions. For the rest of the targets, RT-qPCR was performed using SsoAdvanced Universal SYBR Green Supermix (Bio-Rad) with gene-specific forward and reverse primers at a final concentration of 0.4 μM each (Table 1).

For both SYBR Green and TaqMan assays, RT-qPCR was run under a "Fast" cycling program: initial denaturation at 95°C for 20 s, followed by 40 cycles of 95°C for 1 s and 60°C for 20 s. For SYBR reactions, a melt curve analysis was included. Gene expression was normalized to *RPS18* (human cell-derived samples) or *rps18*/*Gapdh* (C2C12 cell-derived samples) using the ΔΔCt method.

Immunoblotting

For immunoblotting, proteins were extracted using radioimmunoprecipitation assay (RIPA) buffer (Thermo Scientific) supplemented with cOmplete Mini EDTA-free protease inhibitor cocktail (Roche, Mannheim, Germany), following previously described methods with slight modifications.[24,85] Protein concentrations were determined using the Pierce BCA Protein Assay Kit (Thermo Scientific).

Protein samples (2.5 or 5.0 μg) were mixed with NuPAGE LDS Sample Buffer and NuPAGE Sample Reducing Agent (Invitrogen), heated at 70°C for 10 min, and resolved by SDS-PAGE on NuPAGE 3%–8% Tris-Acetate Midi gels (Invitrogen) at 150 V for 70 min. Proteins were transferred onto polyvinylidene fluoride (PVDF) membranes (Millipore, Tullagreen, Ireland) using a semi-dry transfer system at 20 V for 55 min. Membranes were blocked in PBS containing 0.05% Tween 20 (PBS-T) and 5% skim milk with gentle shaking and incubated overnight at 4°C with the following primary antibodies diluted in blocking buffer: anti-activin receptor type IA [EPR4076(2)] (ab155981; Abcam, Cambridge, UK; 1:4,000), anti-GAPDH (14C10) Rabbit mAb (#2118; Cell Signaling Technology, MA, USA; 1:8,000), or anti-V5 Tag monoclonal antibody (SV5-Pk1, R960-25, Invitrogen; 1:4,000). The next day, membranes were incubated with either HRP-conjugated goat anti-mouse IgG (H + L) or goat anti-rabbit IgG (H + L) secondary antibodies (Invitrogen; 1:8,000) for 1 h at room temperature. Following PBS-T washes, protein bands were visualized using ECL Select Detection Reagent (GE Healthcare) and captured on a ChemiDoc Imaging System (Bio-Rad). Densitometric quantification was performed using ImageLab 6.0.1 software (Bio-Rad).

Alkaline phosphatase staining and activity quantification

24 h post-transfection, C2C12 cells were cultured for an additional 4 days in medium supplemented with recombinant human Activin A (rhActivin A, 100 ng/mL; R&D Systems). Prior to the staining experiment, the culture medium was aspirated and stored separately for downstream quantification assays. Cells were subsequently fixed with 4% paraformaldehyde at room temperature and stained using the Alkaline Phosphatase Detection Kit (EMD Millipore) according to the manufacturer's instructions with slight modifications. Briefly, the C2C12 myoblasts ectopically expressing V5-tagged $ACVR1^{WT}$ or $ACVR1^{R206H}$ constructs were seeded onto each well of a 4-chamber

Table 1. Primer sequences for SYBR-based RT-qPCR experiments

Sl	Gene target	Forward primer (5′→3′)	Reverse primer (5′→3′)
1	*ANP32B*	CCATGTAGTCCCTCTTGGTAATC	GACTCACCAATCACAGCTATCC
2	*ACSF3*	ACACGTACAGGGAGCTTTATTC	GTTAGCGCATAGGAAGGAGAC
3	*BCL11B*	CCTGTGGCCAGTGTCAAAT	GTCATAGCAGGCACCCAAG
4	*CEMP1*	CCCAGACCATCCTATCTCTTTG	GCTCTGCCACTGTTCTCTT
5	*CYB561D1*	CCTGGAGGTAGGTCTGGTT	AGCGCTGTCAGAAAGATGG
6	*EXTL3*	TTACCACGCATGGGACATC	CTTGTGAAAGAAGGCAGCAC
7	*FERMT2*	CATGGCGGACAGTTCTTACA	GTCGTGATCTGCTCTGGTATT
8	*HEBP2*	AGTGAGTCTACCATTACCATTTCC	CACAGTCATTTCGGCTCTATCT
9	*P54nrb*	CCTGGCTCCTTTGAGTATGAAT<br>CGCTGGAAGGCACTCATT	TCTCCATCTCCAGCTTCTCA<br>GCAGCTTCCATCTCCATCTC
10	*PIGG*	TCCAAAGCACGTCCAACA	TTCCTTCCACAACTGGGAATAG
11	*PSF*	CTGTTGCTAAGGGCGTAGAC<br>GGCTGAACCAAGTCGTCAT	CACGAATCTTGCTCGGATACTT<br>CCCTTAGCAACAGCATCAATAATC
12	*ZNHIT6*	GTCTCCTAGACAATTTGAGGAACA	CTTGGTGAAGAACTTTCATGTCATT
13	*Havcr1*	GAGAGTGACAGTGGTCTGTATTG<br>CAGGAAGACCCACGACTATTTC	CCTTGTAGTTGTGGGTCTTCTT<br>TTGTGAGTCCATGTGTGTGTAG
14	*Lcn2*	AACTGAATGGGTGGTGAGTG<br>GCCAGTTCACTCTGGGAAATA	TCTCTGGCAACAGGAAAGATG<br>ATGGCGAACTGGTTGTAGTC
15	*Hmox1*	ACAGAGGAACACAAAGACCAG<br>GTTCAAACAGCTCTATCGTGC	GTGTCTGGGATGAGCTAGTG<br>TCTTTGTGTTCCTCTGTCAGC
16	*Tnfa*	CTTCTGTCTACTGAACTTCGGG<br>AGACCCTCACACTCAGATCA	CAGGCTTGTCACTCGAATTTTG<br>TGTCTTTGAGATCCATGCCG
17	*Tgfb*	GGTGGTATACTGAGACACCTTG<br>CGAAGCGGACTACTATGCTAAA	CCCAAGGAAAGGTAGGTGATAG<br>TCTTTGTGTTCCTCTGTCAGC
18	*Gapdh*	TCCATGACAACTTTGGCATTG<br>CATCATCCCTGCATCCACTG	TCACGCCACAGCTTTCCA<br>TCCCGAATGTCTGACGTATTG

Nunc Lab-Tek Chamber Slide System (Thermo Scientific) at a density of 50,000 cells/cm^2. After rhActivin A stimulation, cells were washed with PBS, fixed with cold 100% methanol for 5 min, and air-dried. The alkaline phosphatase staining was performed using a freshly prepared solution of Fast Red Violet, naphthol AS-BI phosphate, and deionized water in a 2:1:1 ratio, incubated for 15 min at room temperature, then rinsed with PBS-T. The chamber wells were detached, and coverslips were mounted with VectaMount AQ. Slides were visualized using a light microscope (B290 TB, Optika, Italy).

The aspirated media collected before fixation were used to assess alkaline phosphatase activity using the QUANTI-Blue system (InvivoGen). Media samples were filtered through 0.45 μm filters and incubated at 56°C for 30 min to inactivate enzyme activity. QUANTI-Blue working solution was made by mixing 1 mL of QB reagent, 1 mL of QB buffer, and 98 mL of sterile water, incubated for 10 min at room temperature. 180 μL of this solution was added to each well of a 96-well plate, followed by 20 μL of each media sample. After incubating at 37°C for an hour, alkaline phosphatase activity was quantified by measuring optical density at 620 nm using a SpectraMax M3 multi-mode microplate reader (Molecular Devices).

ARS staining

ARS staining was performed using the Alizarin Red S Staining Quantification Assay Kit (ScienCell Research Laboratories, Carlsbad, CA) following the manufacturer's protocol with modifications to optimize consistency.

Briefly, 24 h post-transfection, C2C12 cells in a 24-well plate were maintained for an additional 21 days in rhActivin A (R&D Systems) supplemented in osteogenic media containing 50 μg/mL ascorbic acid (Millipore Sigma), and 10 mM β-glycerophosphate (Thermo Scientific Chemicals) in growth media. At the end of this course, cells were washed three times with PBS and fixed in freshly prepared 4% paraformaldehyde for 10 min at room temperature. Fixed cells were then washed three times with deionized water and stained with 40 mM ARS solution (pH 4.2) for 20 min with gentle agitation. Following staining, cells were washed thoroughly three times with deionized water to remove unbound dye and air-dried completely. Cells were visualized using a light microscope (B290 TB, Optika, Italy).

For quantification, 400 μL of 10% acetic acid was added to each well, and the cells were incubated for 30 min at room temperature with

gentle shaking. Cells were then scraped, transferred to 1.5 mL microcentrifuge tubes, vortexed vigorously for 30 s, and heated at 85°C for 10 min to solubilize bound dye. Following heating, samples were immediately placed on ice for 5 min, then centrifuged at 20,000 g for 15 min at 4°C. The supernatant (200 μL) was transferred to a fresh tube, and 75 μL of 10% ammonium hydroxide was added to neutralize the solution. Absorbance was measured at 405 nm using a SpectraMax M3 Multi-Mode Microplate Reader. Mineralization levels were calculated based on the absorbance values, normalized to control wells.

Cell-level vitality, cytotoxicity, and apoptosis assessment

Cell vitality, cytotoxicity, and apoptosis were assessed using the ApoTox-Glo Triplex Assay Kit (Promega, Madison, WI, USA) according to the manufacturer's instructions. Briefly, GM00513 and GM23815 cells were seeded at a density of 50,000 cells/cm^2 onto 96-well flat-bottom microplates (BioLite/Thermo Scientific, Rochester, NY, USA) and allowed to adhere as described in the previous section. To evaluate cell viability and cytotoxicity, 20 μL of a viability/cytotoxicity reagent was added to each well. This reagent contains glycyphenylalanyl-aminofluorocoumarin (GF-AFC) for viability detection and bis-alanylalanyl-phenylalanyl-rhodamine 110 (AAF-R110) for cytotoxicity detection. After reagent addition, plates were placed on an agitator at 180 rpm for 30 s and incubated at 37°C for 40 min. Fluorescence was measured using a SpectraMax M3 Multi-Mode Microplate Reader (Molecular Devices, San Jose, CA, USA) at excitation/emission wavelengths of 400/505 nm (vitality) and 485/520 nm (cytotoxicity). For apoptosis detection, 100 μL of Caspase-Glo 3/7 reagent was added to each well. Plates were agitated at 180 rpm for 30 s and incubated at room temperature for 40 min before luminescence was measured on the same microplate reader. Caspase-3/7 activity served as a surrogate for apoptotic induction. We did not use an exogenous positive control in these assays and interpreted all ApoTox-Glo readouts as relative differences between gapmer treatments.

Mouse husbandry, injections, and sampling

All wild-type C57BL/6 (B6) mice (Jackson Laboratory, USA) were housed in individually ventilated cages at the Health Sciences Laboratory Animal Services (HSLAS), University of Alberta, under a 12 h light/dark cycle with *ad libitum* access to standard chow and water. Retro-orbital (r.o.) injections were performed using insulin syringes with a maximum volume of 95 μL. Cage-mate mice were randomly assigned to treatment groups, and the researcher performing injections was blinded to group allocation. Control mice received an equivalent volume of PBS. Mice were euthanized at experimental endpoints, and blood was collected via cardiac puncture and kept at 4°C for 1 h before serum isolation by centrifugation at 4°C. Urine was collected by bladder massage and was also centrifuged similarly to remove debris and particulates. Skeletal muscles and organs were mounted in tragacanth gum, snap-frozen in liquid nitrogen-cooled isopentane and stored at −80°C until further analysis, as described previously.[86]

ELISA-based quantification of gapmer uptake

For gapmer quantification, 20–30 sections (20 μm thick) of each tissue, or one fine-chopped Achilles tendon per sample, were used for protein extraction in RIPA buffer (Thermo Scientific) supplemented with cOmplete Mini EDTA-free protease inhibitors (Roche, Mannheim, Germany). Gapmer concentrations were measured using a hybridization-based non-competitive ELISA adapted from Yu et al. (2002) with slight modifications.[87] Briefly, diluted extracts and standards were incubated in hybridization buffer (60 mM Na_2HPO_4, 0.9 M NaCl, pH 7.4, 0.24% Tween 20) containing 0.025–0.05 μM of biotinylated template probe (Integrated DNA Technologies) complementary to the gapmer sequence, with an additional 9-base 5′ overhang (GAATAGCGA). After 1 h at 37°C, mixtures were transferred into NeutrAvidin-coated 96-well plates (Thermo Scientific) and incubated for 30 min at 37°C. Plates were washed three times with wash buffer (50 mM Tris-HCl, 150 mM NaCl, pH 7.6, 0.1% Tween 20) and twice with Milli-Q water. A ligation probe (5′-phosphorylated, 3′-digoxigenin labeled) complementary to the overhang sequence was added at 0.067 μM in 1× One-Phor-All Plus buffer (New England Biolabs, MA, USA) containing 400 U/mL T4 ligase (New England Biolabs) and 0.05 mM ATP (New England Biolabs). Ligation was carried out for 2 h at room temperature.

Post-ligation, the wells were washed and incubated with alkaline phosphatase-conjugated anti-digoxigenin antibody (1:5000, Sigma-Aldrich, St. Louis, MO, USA) diluted in SuperBlock blocking buffer (Thermo Scientific) containing 0.0025% Tween 20 for 30 min at 37°C. AttoPhos substrate (Promega) was added, and fluorescence was measured after 30 min at 450 nm excitation/580 nm emission using a SpectraMax M3 reader. Gapmer concentrations were determined from a standard curve.

Serum biochemistry profiling and urinary KIM-1 assessment

Serum samples were analyzed commercially by IDEXX BioAnalytics (California, USA) to assess hepatic, renal, and muscular toxicity markers. Parameters included alkaline phosphatase (ALP), ALT, AST, BUN, creatinine, CPK, T.Bil, T.Prot, albumin, and globulin levels. For the urinary kidney injury molecule-1 (KIM-1) assessment, urine samples were collected from the mice at the experimental endpoint. Urinary KIM-1 quantification was performed using a single-wash, 90-min sandwich ELISA assay according to the manufacturer's protocol (ab213477; Abcam).

Histology

Frozen muscle samples were cryosectioned (5–7 μm) onto poly-L-lysine-coated slides and thawed at room temperature for 30 min. Sections were stained with Mayer's hematoxylin (Electron Microscopy Sciences) for 15 min, rinsed gently under running tap water for 15 min, and counterstained with eosin Y (Electron Microscopy Sciences) for 10 min. Slides were subsequently dehydrated through a graded ethanol series (50%–99%), cleared with a xylene substitute (Thermo Fisher), and mounted using Permount (Fisher Chemical). Blinded investigators performed all analyses.

Statistical analysis

All statistical analyses were performed using GraphPad Prism version 9.0.1 (GraphPad Software, La Jolla, CA, USA). Group comparisons were performed using unpaired one-way ANOVA followed by Tukey's (comparing every group mean with every other group mean) or Dunnett's (comparing multiple treatment groups against a single designated control group) multiple comparisons tests as appropriate. The normality of residuals was assessed using the Shapiro-Wilk test, and homogeneity of variances was evaluated. Statistical significance was determined at α-levels of 0.05, 0.01, 0.001, and 0.0001 ($p = 0.05$, $p = 0.01$, $p = 0.001$, and $p = 0.0001$, respectively). For experiments with genotype and treatment factors, we performed separate one-way ANOVA tests at each genotype or time point. For experiments evaluating responses across distinct genotypes or independent time points, separate one-way ANOVAs were conducted within each specific strata to prevent confounding multifactorial interactions.

DATA AND CODE AVAILABILITY

The datasets generated and analyzed during the current study are available from the corresponding author upon reasonable request. Any additional information required to interpret, verify, or extend the findings of this study can be obtained by contacting the corresponding author.

ACKNOWLEDGMENTS

We would like to express our sincere gratitude to Rohini Roy Roshmi (Department of Pediatrics, University of Alberta) and Stanley Woo (Department of Agricultural, Life, and Environmental Sciences, University of Alberta) for their invaluable assistance with immunoblotting experiments and animal handling. V5-tagged human *ACVR1* constructs were kindly provided by Dr. Takenobu Katagiri (Research Center for Genomic Medicine, Saitama Medical University). Our thanks also go to Dr. Fred B. Berry (Department of Surgery, University of Alberta) for his support with Alizarin Red S staining. We greatly appreciate the insightful advice and technical expertise provided by Drs. Mohammad Nasrullah (Faculty of Pharmacy and Pharmaceutical Sciences, University of Alberta), Daniel Nisakar Meenakshi Sundaram (Department of Chemical and Materials Engineering, University of Alberta), and Hasan Uludağ (Faculties of Pharmacy and Pharmaceutical Sciences, Medicine and Dentistry, and Engineering, University of Alberta) in the execution of *in vitro* toxicity experiments. We also extend our deepest thanks to Drs. Peter Kannu (Department of Medical Genetics, University of Alberta) and Daniel Graf (Department of Oral Biological and Medical Sciences, University of British Columbia) for their exceptional intellectual guidance and support throughout the course of this work. This study was supported by research grants from BC Children's Hospital Foundation/Rare Disease Foundation (18–13), Canadian FOP Network, Canadian Institutes of Health Research (CIHR; #183719), Gilbert K. Winter Fund, and the International FOP Association (IFOPA). T.Y. is supported by Alberta Advanced Education and Technology (AET), Alberta Innovates - Health Solutions (AIHS), Canada Foundation for Innovation, CIHR, Muscular Dystrophy Canada, the University of Alberta Faculty of Medicine and Dentistry, the Friends of Garrett Cumming Research Chair Fund, Henry M. Toupin Neurological Science Research Chair Fund, the U.S. Department of Defense, the National Institutes of Health (NIH), and Women and Children's Health Research Institute (WCHRI). H.M. is supported by WCHRI Postdoctoral Fellowship and Alberta Innovates Postdoctoral Recruitment Fellowships. S.H. and F.M. were recipients of studentship awards from WCHRI. S.A. is supported by the Maternal and Child Health (MatCH) Scholarship, the Alberta Innovates Graduate Student Scholarship (AIGSS), the WCHRI Graduate Studentship, the Andrew Stewart Memorial Graduate Prize, the Alberta Graduate Excellence Scholarship (AGES), and the Friends of Faculty of Medicine and Dentistry Scholarships.

AUTHOR CONTRIBUTIONS

Conceptualization, S.A., R.M., and T.Y.; design, S.A., R.M., and T.Y.; funding acquisition, R.M., and T.Y.; data curation, S.A., S.H., and H.M.; investigation, S.A., S.H., F.M., and H.M.; formal analysis, S.A., H.M., and T.Y.; validation, S.A., H.M., and R.M.; visualization, S.A.; project administration, S.A., R.M., and T.Y.; supervision, S.A., R.M., and T.Y.; resources, S.A. and T.Y.; software, S.A. and T.Y.; writing – original draft, S.A.; writing – editing and revisions, S.A. and T.Y.

DECLARATION OF INTERESTS

T.Y. and R.M. are cofounders of OligomicsTx. T.Y. and R.M. are inventors on patent applications related to allele-selective LNA gapmers targeting $ACVR1^{R206H}$ for the treatment of fibrodysplasia ossificans progressiva (U.S. Provisional Patent Application Serial No. 63/285,547, filed December 3, 2021; International Application No. PCT/CA2022/051756, filed December 1, 2022).

SUPPLEMENTAL INFORMATION

Supplemental information can be found online at https://doi.org/10.1016/j.omtn.2026.102937.

REFERENCES

1. Kaplan, F.S., Glaser, D.L., Shore, E.M., Deirmengian, G.K., Gupta, R., Delai, P., Morhart, R., Smith, R., Le Merrer, M., Rogers, J.G., et al. (2005). The phenotype of fibrodysplasia ossificans progressiva. Clin. Rev. Bone Miner. Metab. *3*, 183–188. https://doi.org/10.1385/BMM:3:3-4:183.
2. Kaplan, F.S., Chakkalakal, S.A., and Shore, E.M. (2012). Fibrodysplasia ossificans progressiva: mechanisms and models of skeletal metamorphosis. Dis. Model. Mech. *5*, 756–762. https://doi.org/10.1242/dmm.010280.
3. Kaplan, F.S., Shen, Q., Lounev, V., Seemann, P., Groppe, J., Katagiri, T., Pignolo, R.J., and Shore, E.M. (2008). Skeletal metamorphosis in fibrodysplasia ossificans progressiva (FOP). J. Bone Miner. Metab. *26*, 521–530. https://doi.org/10.1007/s00774-008-0879-8.
4. Anwar, S., and Yokota, T. (2023). Navigating the Complex Landscape of Fibrodysplasia Ossificans Progressiva: From Current Paradigms to Therapeutic Frontiers. Genes (Basel) *14*, 2162. https://doi.org/10.3390/genes14122162.
5. Kaplan, F.S., Le Merrer, M., Glaser, D.L., Pignolo, R.J., Goldsby, R.E., Kitterman, J.A., Groppe, J., and Shore, E.M. (2008). Fibrodysplasia ossificans progressiva. Best Pract. Res. Clin. Rheumatol. *22*, 191–205. https://doi.org/10.1016/j.berh.2007.11.007.
6. Verma, A.K., Aga, P., Singh, S.K., and Singh, R. (2012). The stone man disease: fibrodysplasia ossificans progressiva: imaging revisited. BMJ Case Rep. *2012*, bcr2012006422. https://doi.org/10.1136/bcr-2012-006422.
7. Shore, E.M., Xu, M., Feldman, G.J., Fenstermacher, D.A., Cho, T.J., Choi, I.H., Connor, J.M., Delai, P., Glaser, D.L., LeMerrer, M., et al. (2006). A recurrent mutation in the BMP type I receptor ACVR1 causes inherited and sporadic fibrodysplasia ossificans progressiva. Nat. Genet. *38*, 525–527. https://doi.org/10.1038/ng1783.
8. Hüning, I., and Gillessen-Kaesbach, G. (2014). Fibrodysplasia ossificans progressiva: Clinical course, genetic mutations and genotype-phenotype correlation. Mol. Syndromol *5*, 201–211. https://doi.org/10.1159/000365770.
9. Dey, D., Bagarova, J., Hatsell, S.J., Armstrong, K.A., Huang, L., Ermann, J., Vonner, A.J., Shen, Y., Mohedas, A.H., Lee, A., et al. (2016). Two tissue-resident progenitor lineages drive distinct phenotypes of heterotopic ossification. Sci. Transl. Med. *8*, 366ra163. https://doi.org/10.1126/scitranslmed.aaf1090.
10. Lees-Shepard, J.B., Yamamoto, M., Biswas, A.A., Stoessel, S.J., Nicholas, S.A.E., Cogswell, C.A., Devarakonda, P.M., Schneider, M.J., Cummins, S.M., Legendre, N.P., et al. (2018). Activin-dependent signaling in fibro/adipogenic progenitors causes fibrodysplasia ossificans progressiva. Nat. Commun. *9*, 471. https://doi.org/10.1038/s41467-018-02872-2.
11. Kaplan, F.S., Mukaddam, M.A., Baujat, G., Brown, M., Cali, A., Cho, T.-J., Crowe, C., Cunto, C.L.D., Delai, P., Diecidue, R.J., et al. (2024). the Medical Management of Fibrodysplasia Ossificans Progressiva: Current Treatment Considerations (Update: July 2024). Proc Intl Clin Council FOP, 1–159. https://assets.nationbuilder.com/ifopa/pages/1042/attachments/original/1721244354/FOP-GUIDELINES-FINAL-2024.pdf?1721244354.
12. Newton, M.C., Allen, P.W., and Ryan, D.C. (1990). Fibrodysplasia ossificans progressiva. Br. J. Anaesth. *64*, 246–250. https://doi.org/10.1093/bja/64.2.246.
13. Aykul, S., Huang, L., Wang, L., Das, N.M., Reisman, S., Ray, Y., Zhang, Q., Rothman, N., Nannuru, K.C., Kamat, V., et al. (2022). Anti-ACVR1 antibodies exacerbate

heterotopic ossification in fibrodysplasia ossificans progressiva (FOP) by activating FOP-mutant ACVR1. J. Clin. Invest. *132*, e153792. https://doi.org/10.1172/JCI153792.

14. Williams, E., Bagarova, J., Kerr, G., Xia, D.-D., Place, E.S., Dey, D., Shen, Y., Bocobo, G.A., Mohedas, A.H., Huang, X., et al. (2021). Saracatinib is an efficacious clinical candidate for fibrodysplasia ossificans progressiva. JCI insight *6*, e95042. https://doi.org/10.1172/jci.insight.95042.

15. Yang, Y.-S., Kim, J.-M., Xie, J., Chaugule, S., Lin, C., Ma, H., Hsiao, E., Hong, J., Chun, H., Shore, E.M., et al. (2022). Suppression of heterotopic ossification in fibrodysplasia ossificans progressiva using AAV gene delivery. Nat. Commun. *13*, 6175. https://doi.org/10.1038/s41467-022-33956-9.

16. Shaikh, U., Khan, A., Kumari, P., Ishfaq, A., Ekhator, C., Yousuf, P., Halappa Nagaraj, R., Raza, H., Ur Rehman, U., Zaman, M.U., et al. (2023). Novel Therapeutic Targets for Fibrodysplasia Ossificans Progressiva: Emerging Strategies and Future Directions. Cureus *15*, e42614. https://doi.org/10.7759/cureus.42614.

17. Wentworth, K.L., Masharani, U., and Hsiao, E.C. (2019). Therapeutic advances for blocking heterotopic ossification in fibrodysplasia ossificans progressiva. Br. J. Clin. Pharmacol. *85*, 1180–1187. https://doi.org/10.1111/bcp.13823.

18. U.S. Food and Drug Administration (FDA) (2023). FDA approves first treatment for Fibrodysplasia Ossificans Progressiva. https://www.fda.gov/drugs/news-events-human-drugs/fda-approves-first-treatment-fibrodysplasia-ossificans-progressiva.

19. Takahashi, M., Katagiri, T., Furuya, H., and Hohjoh, H. (2012). Disease-causing allele-specific silencing against the ALK2 mutants, R206H and G356D, in fibrodysplasia ossificans progressiva. Gene Ther. *19*, 781–785. https://doi.org/10.1038/gt.2011.193.

20. Kaplan, J., Kaplan, F.S., and Shore, E.M. (2012). Restoration of normal BMP signaling levels and osteogenic differentiation in FOP mesenchymal progenitor cells by mutant allele-specific targeting. Gene Ther. *19*, 786–790. https://doi.org/10.1038/gt.2011.152.

21. Gu, Z., Reynolds, E.M., Song, J., Lei, H., Feijen, A., Yu, L., He, W., MacLaughlin, D.T., Van Den Eijnden-Van Raaij, J., Donahoe, P.K., and Li, E. (1999). The type I serine/threonine kinase receptor ActRIA (ALK2) is required for gastrulation of the mouse embryo. Development *126*, 2551–2561. https://doi.org/10.1242/dev.126.11.2551.

22. Mishina, Y., Crombie, R., Bradley, A., and Behringer, R.R. (1999). Multiple roles for activin-like kinase-2 signaling during mouse embryogenesis. Dev. Biol. *213*, 314–326. https://doi.org/10.1006/dbio.1999.9378.

23. Dudas, M., Sridurongrit, S., Nagy, A., Okazaki, K., and Kaartinen, V. (2004). Craniofacial defects in mice lacking BMP type I receptor Alk2 in neural crest cells. Mech. Dev. *121*, 173–182. https://doi.org/10.1016/j.mod.2003.12.003.

24. Maruyama, R., Nguyen, Q., Roshmi, R.R., Touznik, A., and Yokota, T. (2022). Allele-Selective LNA Gapmers for the Treatment of Fibrodysplasia Ossificans Progressiva Knock Down the Pathogenic ACVR1^{R206H} Transcript and Inhibit Osteogenic Differentiation. Nucleic Acid Ther. *32*, 185–193. https://doi.org/10.1089/nat.2021.0009.

25. Crooke, S.T. (1999). Molecular mechanisms of action of antisense drugs. Biochim. Biophys. Acta *1489*, 31–44. https://doi.org/10.1016/S0167-4781(99)00148-7.

26. Anwar, S., Mir, F., and Yokota, T. (2023). Enhancing the Effectiveness of Oligonucleotide Therapeutics Using Cell-Penetrating Peptide Conjugation, Chemical Modification, and Carrier-Based Delivery Strategies. Pharmaceutics *15*, 1130. https://doi.org/10.3390/pharmaceutics15041130.

27. Khvorova, A., and Watts, J.K. (2017). The chemical evolution of oligonucleotide therapies of clinical utility. Nat. Biotechnol. *35*, 238–248. https://doi.org/10.1038/nbt.3765.

28. Swayze, E.E., and Bhat, B. (2007). The medicinal chemistry of oligonucleotides. In Antisense Drug Technology: Principles, Strategies, and Applications, Second Edition, S.T. Crooke, ed. (CRC Press)), pp. 143–182. https://doi.org/10.1201/9780849387951.ch6.

29. Frazier, K.S. (2015). Antisense Oligonucleotide Therapies:The Promise and the Challenges from a Toxicologic Pathologist's Perspective. Toxicol. Pathol. *43*, 78–89. https://doi.org/10.1177/0192623314551840.

30. Swayze, E.E., Siwkowski, A.M., Wancewicz, E.V., Migawa, M.T., Wyrzykiewicz, T.K., Hung, G., Monia, B.P., and Bennett, C.F. (2007). Antisense oligonucleotides containing locked nucleic acid improve potency but cause significant hepatotoxicity in animals. Nucleic Acids Res. *35*, 687–700. https://doi.org/10.1093/nar/gkl1071.

31. Burdick, A.D., Sciabola, S., Mantena, S.R., Hollingshead, B.D., Stanton, R., Warneke, J.A., Zeng, M., Martsen, E., Medvedev, A., Makarov, S.S., et al. (2014). Sequence motifs associated with hepatotoxicity of locked nucleic acid–modified antisense oligonucleotides. Nucleic Acids Res. *42*, 4882–4891. https://doi.org/10.1093/nar/gku142.

32. Kakiuchi-Kiyota, S., Koza-Taylor, P.H., Mantena, S.R., Nelms, L.F., Enayetallah, A.E., Hollingshead, B.D., Burdick, A.D., Reed, L.A., Warneke, J.A., Whiteley, L.O., et al. (2014). Comparison of hepatic transcription profiles of locked ribonucleic acid antisense oligonucleotides: Evidence of distinct pathways contributing to non-target mediated toxicity in mice. Toxicol. Sci. *138*, 234–248. https://doi.org/10.1093/toxsci/kft278.

33. Kamola, P.J., Kitson, J.D.A., Turner, G., Maratou, K., Eriksson, S., Panjwani, A., Warnock, L.C., Douillard Guilloux, G.A., Moores, K., Koppe, E.L., et al. (2015). *In silico* and *in vitro* evaluation of exonic and intronic off-target effects form a critical element of therapeutic ASO gapmer optimization. Nucleic Acids Res. *43*, 8638–8650. https://doi.org/10.1093/nar/gkv857.

34. Kasuya, T., Hori, S.I., Watanabe, A., Nakajima, M., Gahara, Y., Rokushima, M., Yanagimoto, T., and Kugimiya, A. (2016). Ribonuclease H1-dependent hepatotoxicity caused by locked nucleic acid-modified gapmer antisense oligonucleotides. Sci. Rep. *6*, 30377. https://doi.org/10.1038/srep30377.

35. Burel, S.A., Hart, C.E., Cauntay, P., Hsiao, J., Machemer, T., Katz, M., Watt, A., Bui, H.H., Younis, H., Sabripour, M., et al. (2016). Hepatotoxicity of high affinity gapmer antisense oligonucleotides is mediated by RNase H1 dependent promiscuous reduction of very long pre-mRNA transcripts. Nucleic Acids Res. *44*, 2093–2109. https://doi.org/10.1093/nar/gkv1210.

36. Kakiuchi-Kiyota, S., Whiteley, L.O., Ryan, A.M., and Mathialagan, N. (2016). Development of a method for profiling protein interactions with LNA-modified antisense oligonucleotides using protein microarrays. Nucleic Acid Ther. *26*, 93–101. https://doi.org/10.1089/nat.2015.0576.

37. Kamola, P.J., Maratou, K., Wilson, P.A., Rush, K., Mullaney, T., McKevitt, T., Evans, P., Ridings, J., Chowdhury, P., Roulois, A., et al. (2017). Strategies for *In Vivo* Screening and Mitigation of Hepatotoxicity Associated with Antisense Drugs. Mol. Ther. Nucleic Acids *8*, 383–394. https://doi.org/10.1016/j.omtn.2017.07.003.

38. Dieckmann, A., Hagedorn, P.H., Burki, Y., Brügmann, C., Berrera, M., Ebeling, M., Singer, T., and Schuler, F. (2018). A Sensitive *In Vitro* Approach to Assess the Hybridization-Dependent Toxic Potential of High Affinity Gapmer Oligonucleotides. Mol. Ther. Nucleic Acids *10*, 45–54. https://doi.org/10.1016/j.omtn.2017.11.004.

39. Bohr, H.G., Shim, I., Stein, C., Ørum, H., Hansen, H.F., and Koch, T. (2017). Electronic Structures of LNA Phosphorothioate Oligonucleotides. Mol. Ther. Nucleic Acids *8*, 428–441. https://doi.org/10.1016/j.omtn.2017.05.011.

40. Shen, W., De Hoyos, C.L., Sun, H., Vickers, T.A., Liang, X.H., and Crooke, S.T. (2018). Acute hepatotoxicity of 2 fluoro-modified 5–10–5 gapmer phosphorothioate oligonucleotides in mice correlates with intracellular protein binding and the loss of DBHS proteins. Nucleic Acids Res. *46*, 2204–2217. https://doi.org/10.1093/nar/gky060.

41. Stanton, R., Sciabola, S., Salatto, C., Weng, Y., Moshinsky, D., Little, J., Walters, E., Kreeger, J., Dimattia, D., Chen, T., et al. (2012). Chemical modification study of antisense gapmers. Nucleic Acid Ther. *22*, 344–359. https://doi.org/10.1089/nat.2012.0366.

42. Hagedorn, P.H., Yakimov, V., Ottosen, S., Kammler, S., Nielsen, N.F., Høg, A.M., Hedtjärn, M., Meldgaard, M., Møller, M.R., Ørum, H., et al. (2013). Hepatotoxic potential of therapeutic oligonucleotides can be predicted from their sequence and modification pattern. Nucleic Acid Ther. *23*, 302–310. https://doi.org/10.1089/nat.2013.0436.

43. Shen, W., Liang, X.H., Sun, H., and Crooke, S.T. (2015). 2′-Fluoro-modified phosphorothioate oligonucleotide can cause rapid degradation of P54nrb and PSF. Nucleic Acids Res. *43*, 4569–4578. https://doi.org/10.1093/nar/gkv298.

44. Shen, W., De Hoyos, C.L., Migawa, M.T., Vickers, T.A., Sun, H., Low, A., Bell, T.A., Rahdar, M., Mukhopadhyay, S., Hart, C.E., et al. (2019). Chemical modification of PS-ASO therapeutics reduces cellular protein-binding and improves the therapeutic index. Nat. Biotechnol. *37*, 640–650. https://doi.org/10.1038/s41587-019-0106-2.

45. Crooke, S.T., Wang, S., Vickers, T.A., Shen, W., and Liang, X.-H. (2017). Cellular uptake and trafficking of antisense oligonucleotides. Nat. Biotechnol. *35*, 230–237. https://doi.org/10.1038/nbt.3779.

46. Hino, K., Ikeya, M., Horigome, K., Matsumoto, Y., Ebise, H., Nishio, M., Sekiguchi, K., Shibata, M., Nagata, S., Matsuda, S., and Toguchida, J. (2015). Neofunction of ACVR1 in fibrodysplasia ossificans progressiva. Proc. Natl. Acad. Sci. USA *112*, 15438–15443. https://doi.org/10.1073/pnas.1510540112.

47. Fukuda, T., Kohda, M., Kanomata, K., Nojima, J., Nakamura, A., Kamizono, J., Noguchi, Y., Iwakiri, K., Kondo, T., Kurose, J., et al. (2009). Constitutively activated ALK2 and increased SMAD1/5 cooperatively induce bone morphogenetic protein signaling in fibrodysplasia ossificans progressiva. J. Biol. Chem. *284*, 7149–7156. https://doi.org/10.1074/jbc.M801681200.

48. Song, G.A., Kim, H.J., Woo, K.M., Baek, J.H., Kim, G.S., Choi, J.Y., and Ryoo, H.M. (2010). Molecular consequences of the $ACVR1^{R206H}$ mutation of fibrodysplasia ossificans progressiva. J. Biol. Chem. *285*, 22542–22553. https://doi.org/10.1074/jbc.M109.094557.

49. Bailey, J.K., Shen, W., Liang, X.H., and Crooke, S.T. (2017). Nucleic acid binding proteins affect the subcellular distribution of phosphorothioate antisense oligonucleotides. Nucleic Acids Res. *45*, 10649–10671. https://doi.org/10.1093/nar/gkx709.

50. Vickers, T.A., Rahdar, M., Prakash, T.P., and Crooke, S.T. (2019). Kinetic and subcellular analysis of PS-ASO/protein interactions with P54nrb and RNase H1. Nucleic Acids Res. *47*, 10865–10880. https://doi.org/10.1093/nar/gkz771.

51. Eekhoff, E.M.W., de Ruiter, R.D., Smilde, B.J., Schoenmaker, T., de Vries, T.J., Netelenbos, C., Hsiao, E.C., Scott, C., Haga, N., Grunwald, Z., et al. (2022). Gene Therapy for Fibrodysplasia Ossificans Progressiva: Feasibility and Obstacles. Hum. Gene Ther. *33*, 782–788. https://doi.org/10.1089/hum.2022.023.

52. Doxtader Lacy, K.A., Liang, X.H., Zhang, L., and Crooke, S.T. (2022). RNA modifications can affect RNase H1-mediated PS-ASO activity. Mol. Ther. Nucleic Acids *28*, 814–828. https://doi.org/10.1016/j.omtn.2022.05.024.

53. Hagedorn, P.H., Hansen, B.R., Koch, T., and Lindow, M. (2017). Managing the sequence-specificity of antisense oligonucleotides in drug discovery. Nucleic Acids Res. *45*, 2262–2282. https://doi.org/10.1093/nar/gkx056.

54. Hoy, S.M. (2022). Palovarotene: First Approval. Drugs *82*, 711–716. https://doi.org/10.1007/s40265-022-01709-z.

55. Palovarotene (2023). Am. J. Health Syst. Pharm. *80*, 1763–1767. https://doi.org/10.1093/ajhp/zxad229.

56. International Clinical Council on Fibrodysplasia Ossificans Progressiva (ICCFOP) (2013). Statement Regarding Palovarotene from the International Clinical Council (ICC) on FOP (ICCFOP).

57. Talha, M., and Ali, M.H. (2024). Palovarotene approved as first treatment for fibrodysplasia ossificans progressiva (FOP). J. Rare Dis. *3*, 8. https://doi.org/10.1007/s44162-024-00032-3.

58. Rauner, M., Seefried, L., and Shore, E. (2020). Genetics and future therapy prospects of fibrodysplasia ossificans progressiva. Medizinische Genet. *31*, 391–396. https://doi.org/10.1007/s11825-019-00279-y.

59. Yang, Y.O., Gong, X., Getsy, J., Wang, P., Liu, X., Sheng, J., Chen, X., and Rockich, K. (2025). Pharmacokinetics of Zilurgisertib With and Without Food from Single and Multiple Ascending Dose Phase 1 Studies in Healthy Adults. Eur. J. Drug Metab. Pharmacokinet. *50*, 65–80. https://doi.org/10.1007/s13318-024-00926-z.

60. Wang, D., Tai, P.W.L., and Gao, G. (2019). Adeno-associated virus vector as a platform for gene therapy delivery. Nat. Rev. Drug Discov. *18*, 358–378. https://doi.org/10.1038/s41573-019-0012-9.

61. Tang, A., and Yokota, T. (2025). Is Duchenne gene therapy a suitable treatment despite its immunogenic class effect? Expert Opin. Drug Saf. *24*, 395–411. https://doi.org/10.1080/14740338.2024.2447072.

62. Chamberlain, J.S., Davidson, B.L., George, L.A., Byrne, B.J., and Barrett, D. (2025). The future of gene therapy: Safer vectors, sharper focus. Mol. Ther. *33*, 4694–4695. https://doi.org/10.1016/j.ymthe.2025.08.029.

63. Moldavskii, D., Gilazieva, Z., Fattakhova, A., Solovyeva, V., Issa, S., Sufianov, A., Sufianova, G., and Rizvanov, A. (2025). AAV-Based Gene Therapy: Opportunities, Risks, and Scale-Up Strategies. Int. J. Mol. Sci. *26*, 8282. https://doi.org/10.3390/ijms26178282.

64. Kachanov, A., Kostyusheva, A., Brezgin, S., Karandashov, I., Ponomareva, N., Tikhonov, A., Lukashev, A., Pokrovsky, V., Zamyatnin, A.A., Parodi, A., et al. (2024). The menace of severe adverse events and deaths associated with viral gene therapy and its potential solution. Med. Res. Rev. *44*, 2112–2193. https://doi.org/10.1002/med.22036.

65. Hatsell, S.J., Idone, V., Wolken, D.M.A., Huang, L., Kim, H.J., Wang, L., Wen, X., Nannuru, K.C., Jimenez, J., Xie, L., et al. (2015). $ACVR1^{R206H}$ receptor mutation causes fibrodysplasia ossificans progressiva by imparting responsiveness to activin A. Sci. Transl. Med. *7*. https://doi.org/10.1126/scitranslmed.aac4358.

66. Di Rocco, M., Forleo-Neto, E., Pignolo, R.J., Keen, R., Orcel, P., Funck-Brentano, T., Roux, C., Kolta, S., Madeo, A., Bubbear, J.S., et al. (2023). Garetosmab in fibrodysplasia ossificans progressiva: a randomized, double-blind, placebo-controlled phase 2 trial. Nat. Med. *29*, 2615–2624. https://doi.org/10.1038/s41591-023-02561-8.

67. Wenkert, D., Bhargava, P., and Smith, V. (2024). LB 596 - Inhibition of MMP9 as a novel treatment strategy for Fibrodysplasia Ossificans Progressiva (FOP): Safety analysis of the anti-MMP9 antibody Andecaliximab in development for FOP. In ASBMR Annual Meeting.

68. Davis, A.J., Brooijmans, N., Brubaker, J.D., Stevison, F., LaBranche, T.P., Albayya, F., Fleming, P., Hodous, B.L., Kim, J.L., Kim, S., et al. (2024). An ALK2 inhibitor, BLU-782, prevents heterotopic ossification in a mouse model of fibrodysplasia ossificans progressiva. Sci. Transl. Med. *16*. https://doi.org/10.1126/scitranslmed.abp8334.

69. Shi, S.T., Cai, J., de Gorter, D.J.J., Sanchez-Duffhues, G., Kemaladewi, D.U., Hoogaars, W.M.H., Aartsma-Rus, A., 't Hoen, P.A.C., and ten Dijke, P. (2013). Antisense-Oligonucleotide Mediated Exon Skipping in Activin-Receptor-Like Kinase 2: Inhibiting the Receptor That Is Overactive in Fibrodysplasia Ossificans Progressiva. PLoS One *8*. https://doi.org/10.1371/journal.pone.0069096.

70. Maurya, A., Gaurav, A., Chauhan, V., and Verma, A. (2026). Advances in genetic and pharmacological therapeutic strategies for fibrodysplasia ossificans progressiva. Discov. Med. *3*, 14. https://doi.org/10.1007/s44337-026-00561-4.

71. Hagedorn, P.H., Persson, R., Funder, E.D., Albæk, N., Diemer, S.L., Hansen, D.J., Møller, M.R., Papargyri, N., Christiansen, H., Hansen, B.R., et al. (2018). Locked nucleic acid: modality, diversity, and drug discovery. Drug Discov. Today *23*, 101–114. https://doi.org/10.1016/j.drudis.2017.09.018.

72. Di Martino, M.T., Arbitrio, M., Caracciolo, D., Scionti, F., Tagliaferri, P., and Tassone, P. (2020). Dose-Finding Study and Pharmacokinetics Profile of the Novel 13-Mer Antisense miR-221 Inhibitor in Sprague-Dawley Rats. Mol. Ther. Nucleic Acids *20*, 73–85. https://doi.org/10.1016/j.omtn.2020.01.036.

73. Levin, A.A., Yu, R.Z., and Geary, R.S. (2007). Basic principles of the pharmacokinetics of antisense oligonucleotide drugs. Antisense Drug Technol. Princ. Strateg, Second Ed. (Appl), pp. 183–215. https://doi.org/10.1201/9780849387951.ch7.

74. Geary, R.S. (2009). Antisense oligonucleotide pharmacokinetics and metabolism. Expert Opin. Drug Metab. Toxicol. *5*, 381–391. https://doi.org/10.1517/17425250902877680.

75. Bosgra, S., Sipkens, J., de Kimpe, S., Den Besten, C., Datson, N., and van Deutekom, J. (2019). The Pharmacokinetics of 2'-O-Methyl Phosphorothioate Antisense Oligonucleotides: Experiences from Developing Exon Skipping Therapies for Duchenne Muscular Dystrophy. Nucleic Acid Ther. *29*, 305–322.

76. Fey, R.A., Templin, M.V., McDonald, J.D., Yu, R.Z., Hutt, J.A., Gigliotti, A.P., Henry, S.P., and Reed, M.D. (2014). Local and systemic tolerability of a 2′O-methoxyethyl antisense oligonucleotide targeting interleukin-4 receptor-α delivery by inhalation in mouse and monkey. Inhal. Toxicol. *26*, 452–463. https://doi.org/10.3109/08958378.2014.907587.

77. Crosby, J.R., Zhao, C., Jiang, C., Bai, D., Katz, M., Greenlee, S., Kawabe, H., McCaleb, M., Rotin, D., Guo, S., et al. (2017). Inhaled ENaC antisense oligonucleotide ameliorates cystic fibrosis-like lung disease in mice. J. Cyst. Fibros. *16*, 671–680. https://doi.org/10.1016/j.jcf.2017.05.003.

78. Klabenkova, K., Fokina, A., and Stetsenko, D. (2021). Chemistry of Peptide-Oligonucleotide Conjugates: A Review. Molecules *26*, 5420. https://doi.org/10.3390/molecules26175420.

79. Desjardins, C.A., Picariello, T., Natoli, T.A., Yoder, N., Yao, M., Valdivia, B., Tahaei, E., Johnson, J., Qiu, Q., More, P., et al. (2025). The FORCE^{TM} Platform Achieves Robust and Durable DUX4 Suppression and Improves Muscle Function in a

Facioscapulohumeral Muscular Dystrophy Mouse Model. In Muscular Dystrophy Association (MDA) Clinical & Scientific Conference, pp. P127.

80. Statland, J., Halseth, A., Zhu, Y., Day, J., Johnson, N., Laverty, C., Quan, D., Quinn, C., Subramony, S., Tawil, R., et al. (2024). Interim Results from FORTITUDE™, a Randomized Phase 1/2 Trial Evaluating AOC 1020 in Adults with FSHD. In 2024 FSHD Society International Research Congress.

81. Aslesh, T., Erkut, E., Ren, J., Lim, K.R.Q., Woo, S., Hatlevig, S., Moulton, H.M., Gosgnach, S., Greer, J., Maruyama, R., et al. (2023). DG9-conjugated morpholino rescues phenotype in SMA mice by reaching the CNS via a subcutaneous administration. JCI Insight *8*, e160516. https://doi.org/10.1172/jci.insight.160516.

82. Lim, K.R.Q., Woo, S., Melo, D., Huang, Y., Dzierlega, K., Shah, M.N.A., Aslesh, T., Roshmi, R.R., Echigoya, Y., Maruyama, R., et al. (2022). Development of DG9 peptide-conjugated single- and multi-exon skipping therapies for the treatment of Duchenne muscular dystrophy. Proc. Natl. Acad. Sci. USA *119*. https://doi.org/10.1073/pnas.2112546119.

83. Chwalenia, K., Wood, M.J.A., and Roberts, T.C. (2025). Progress and prospects in antisense oligonucleotide-mediated exon skipping therapies for Duchenne muscular dystrophy. J. Muscle Res. Cell Motil. *46*, 293–300. https://doi.org/10.1007/s10974-024-09688-2.

84. Yoshida, T., Naito, Y., Sasaki, K., Uchida, E., Sato, Y., Naito, M., Kawanishi, T., Obika, S., and Inoue, T. (2018). Estimated number of off-target candidate sites for antisense oligonucleotides in human mRNA sequences. Genes Cells *23*, 448–455. https://doi.org/10.1111/gtc.12587.

85. Anwar, S., and Yokota, T. (2023). Morpholino-Mediated Exons 28–29 Skipping of Dysferlin and Characterization of Multiexon-skipped Dysferlin using RT-PCR, Immunoblotting, and Membrane Wounding Assay. In Muscular Dystrophy Therapeutics. Methods in Molecular Biology, *2587*, Y. Maruyama and T. Yokota, eds. (Human Press), pp. 183–196. https://doi.org/10.1007/978-1-0716-2772-3_11.

86. Anwar, S., and Yokota, T. (2023). Rapid Freezing of Skeletal and Cardiac Muscles Using Isopentane Cooled with Liquid Nitrogen and Tragacanth Gum for Histological, Genetic, and Protein Expression Studies. In Muscular Dystrophy Therapeutics: Methods and Protocols, pp. 45–53. https://doi.org/10.1007/978-1-0716-2772-3_3.

87. Yu, R.Z., Baker, B., Chappell, A., Geary, R.S., Cheung, E., and Levin, A.A. (2002). Development of an Ultrasensitive Noncompetitive Hybridization-Ligation Enzyme-Linked Immunosorbent Assay for the Determination of Phosphorothioate Oligodeoxynucleotide in Plasma. Anal. Biochem. *304*. https://doi.org/10.1006/abio.2002.5576.

OMTN, Volume *37*

Supplemental information

Single-base 2′OMe-modified LNA and MOE gapmers selectively silence *ACVR1*^{R206H} in fibrodysplasia ossificans progressiva

Saeed Anwar, Sarah Hay, Hidenori Moriyama, Farin Mir, Rika Maruyama, and Toshifumi Yokota

Table S1: Predicted off-target candidates for lead antisense gapmers. Potential off-target transcripts were identified at the web interface of GGGenome server (https://gggenome.dbcls.jp), allowing for up to 2 mismatches/indels for MOE gapmers and 1 for LNA gapmers. Alignments show the target gene sequence relative to the designated gapmer sequence. Bold indicates base mismatches, dash (–) denotes deletions, and underline denotes insertions. We searched against the human RefSeq RNA database (release 230, May 2025), for both the sense and antisense strands. *ACVR1*/*ACVR1*^{R206H} is excluded from this list.

Gene	Sequence	Number of mismatches
**MOE3r**	TGG CTC ACC AGA TTA CAC TG	
ANP32B	TGG CT- ACC AG- TTA CAC TG	2
**MOE3s**	TGG CTC ACC AGC TTA CAC TG	
ANP32B	TGG CT- ACC AG- TTA CAC TG	2
**LNA16r**	CAG TGG CTC ACC AGA T	
TEC	CAG TGG CTC -CC AGA T	1
TENM1	CAG -GG CTC ACC AGA T	1
CD163L1	CAG -GG CTC ACC AGA T	1
MTERF4	CAG TGG CTC -CC AGA T	1
IRF2BP1	CAG TGG CTC **G**CC AGA T	1
NFATC3	CAG TGG CT**G** ACC AGA T	1
NCF4	CAG TGG CTC -CC AGA T	1
PCDH17	ATC TGG TGA G-C ACT G	1
SF3B3	ATC TGG TGA G-C ACT G	1
**LNA16s**	CAG TGG CTC ACC AGC T	
CEMP1	CAG -GG CTC ACC AGC T	1
PIGG	CA**C** TGG CTC ACC AGC T	1
EXTL3	CAG -GG CTC ACC AGC T	1
ZNHIT6	CAG TGG CTC A-C AGC T	1
PRICKLE1	CAG TGG C-C ACC AGC T	1
FERMT2	CAG TGG C**A**C ACC AGC T	1
BCL11B	CAG -GG CTC ACC AGC T	1
CD163L1	CAG -GG CTC ACC AGC T	1
MTERF4	CAG TGG CTC ACC AG**G** T	1
ACSF3	C**T**G TGG CTC ACC AGC T	1
HEBP2	CAG TGG CTC A-C AGC T	1
CYP561D1	CAG TGG CTC -CC AGC T	1
STRN	AGC TGG TGA GCC -CT G	1
GALNT14	AGC -GG TGA GCC ACT G	1
TNK2	AGC T-G TGA GCC ACT G	1
ADGRA3	AGC T-G TGA GCC ACT G	1
GALNT17	AGC T-G TGA GCC ACT G	1
CAMTA1	AGC TG**C** TGA GCC ACT G	1
TACC2	AGC TGG TGA GCC CAC TG	1
A1CF	AGC TGG **A**GA GCC ACT G	1
SPPL3	AGC T-G TGA GCC ACT G	1
ESPL1	AGC TGG TG- GCC ACT G	1
AMDHD2	AGC TGG TGA GCC -CT G	1
ATAD2B	AGC T**T**G TGA GCC ACT G	1
ASIC4	AGC TGG TG**G** GCC ACT G	1
ITPKB	AGC TGG -GA GCC ACT G	1
AANAT	AGC -GG TGA GCC ACT G	1
PCDHA9	AGC T-G TGA GCC ACT G	1
NPLOC4	AGC T-G TGA GCC ACT G	1
RPL15	AGC TGG TGA GCC A**G**T G	1
HOGA1	AGC T-G TGA GCC ACT G	1
KIF4B	AGC TGG TGA GCA CAC TG	1
PRDM16	AGC TGG TG- GCC ACT G	1

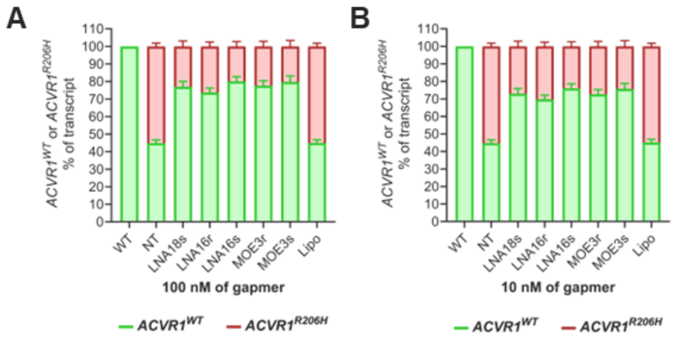

Figure S1. Gapmer treatment increases *ACVR1^{WT}* allelic contribution while reducing *ACVR1^{R206H}* expression in FOP patient-derived fibroblasts.

(**A–B**) Allelic composition of total *ACVR1* transcripts following treatment with 100 nM (**A**) or 10 nM (**B**) gapmers. Green and red bars represent *ACVR1^{WT}* and *ACVR1^{R206H}* transcript fractions, respectively. Data are presented as the mean ± standard error of the mean for the fractions calculated from all replicates. NT, non-treated, Lipo, Lipofectamine 3000 (a commercially available transfection reagent)

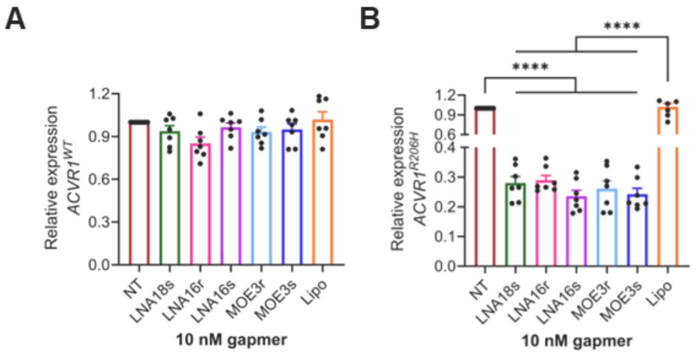

Figure S2. Selective suppression of *ACVR1*^{R206H} in C2C12 cells expressing V5-tagged constructs.

(**A–B**) RT-qPCR quantification of *ACVR1*^{WT} (**A**) and *ACVR1*^{R206H} (**B**) transcripts in C2C12 cells transfected with plasmids encoding V5-tagged *ACVR1*^{WT} and *ACVR1*^{R206H} and treated with 10 nM gapmers for 48 hours. Statistics, one-way ANOVA with Tukey's multiple comparisons test; ****$p<0.0001$. Data are represented as mean ± standard error of the mean. Each black dot represents an individual biological replicate. NT, non-treated, Lipo, Lipofectamine 3000 (a commercially available transfection reagent).

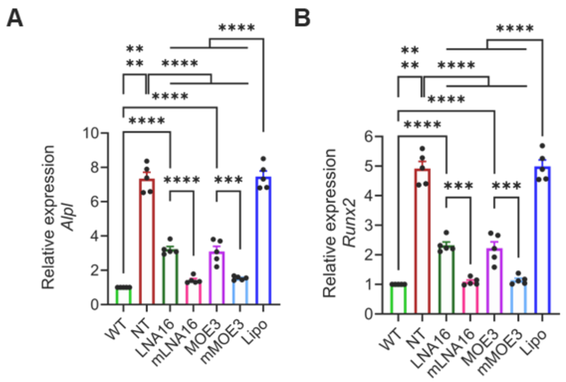

Figure S3. 2′OMe-modified gapmers suppress undesired osteogenic gene expression more effectively than unmodified counterparts.

(**A–B**) RT-qPCR quantification of osteogenic markers *Alpl* (**A**) and *Runx2* (**B**) in C2C12 cells transfected with V5-tagged $ACVR1^{WT}$ or $ACVR1^{R206H}$ and treated with 10 nM gapmers. Data collected 48 hours post-treatment. Statistics, one-way ANOVA with Tukey's multiple comparisons test; ****p<0.0001. Data are represented as mean ± standard error of the mean. Each black dot represents an individual biological replicate. NT, non-treated, Lipo, Lipofectamine 3000 (a commercially available transfection reagent).

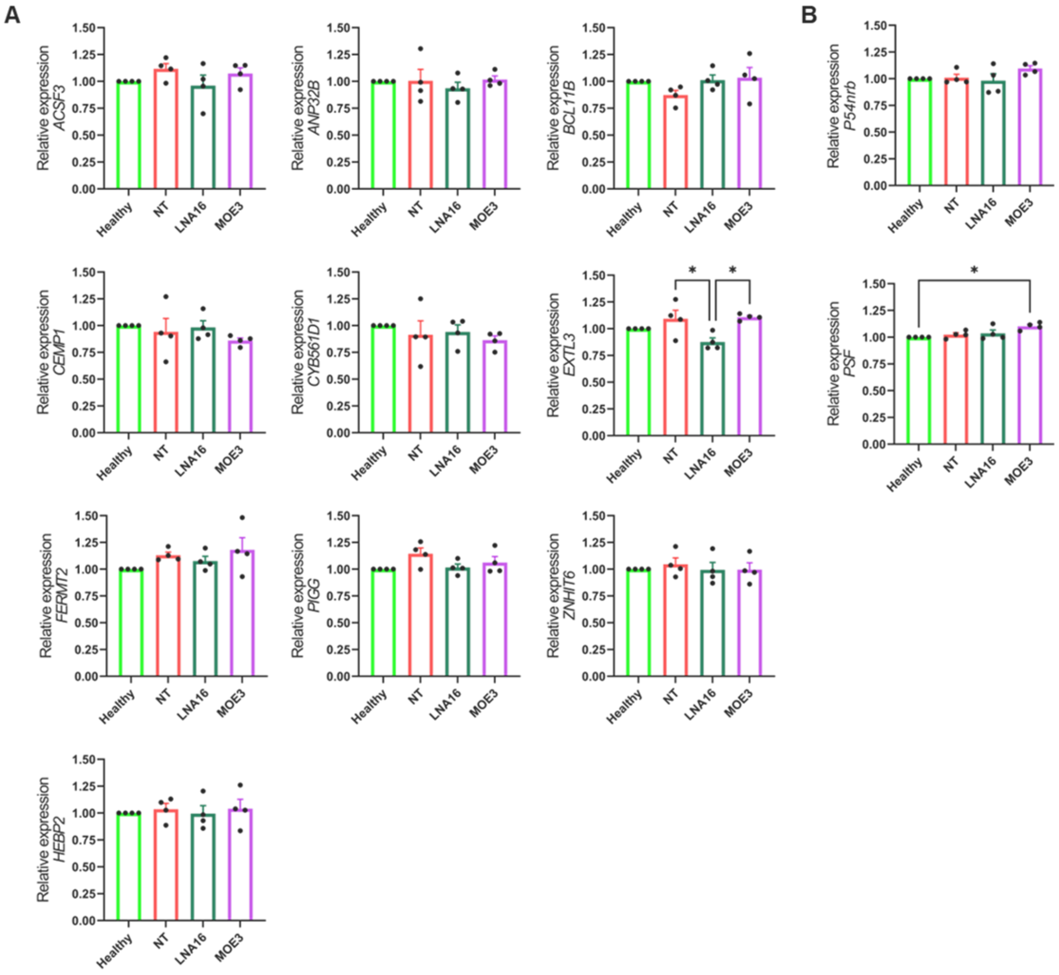

Figure S4. Gapmer treatment does not significantly alter the expression of predicted off-target genes or known ASO-sensitive genes.

(**A–B**) RT-qPCR assessment of the expression of (**A**) predicted off-target candidate genes and (**B**) paraspeckle genes known to be broadly affected by ASO treatments. FOP patient-derived fibroblasts were treated with 10 nM of LNA16 or MOE3 and RNA was harvested 48 hours post-treatment. Each black dot represents an individual biological replicate. Data are presented as the mean ± standard error of the mean. Statistics, one-way ANOVA with Tukey's multiple comparisons test; *p<0.05.

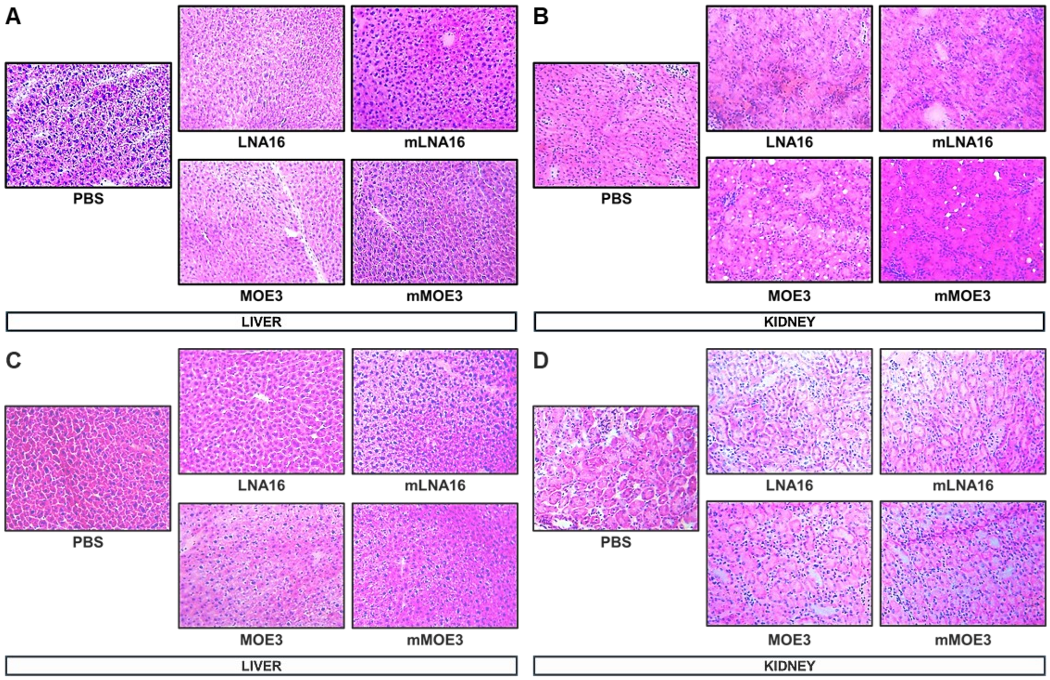

Figure S5: Histopathological analysis of liver and kidney following gapmer administration.

(**A–D**) Representative hematoxylin and eosin-stained sections of (**A, C**) liver and (**B, D**) kidney tissues harvested (**A, B**) 3 days and (**C, D**) 15 days post-injection. At 3 days, liver sections from mice treated with unmodified gapmers, i.e., LNA16 and MOE3, exhibited mild/diffuse hepatocellular rarefaction and microvesicular vacuolation, with no significant evidence of signs of necrosis, apoptosis, or inflammatory infiltrates. Mice receiving 2′OMe-modified gapmers, i.e., mLNA16 and mMOE3, demonstrated largely preserved hepatic architecture. In the kidney at 3 days, unmodified gapmers resulted in some localized proximal tubular epithelial vacuolation and mild luminal dilation. Importantly, signs of acute tubular necrosis or cast formation were not visible. 2′OMe-modified gapmers markedly attenuated these proximal tubular changes. By day 15 post-treatment, tissue architecture across all gapmer-treated groups was almost indistinguishable from PBS controls, confirming that early minor morphological shifts represent a transient, adaptive clearance response rather than progressive toxicologic pathology. Scale bar: 100 μm.

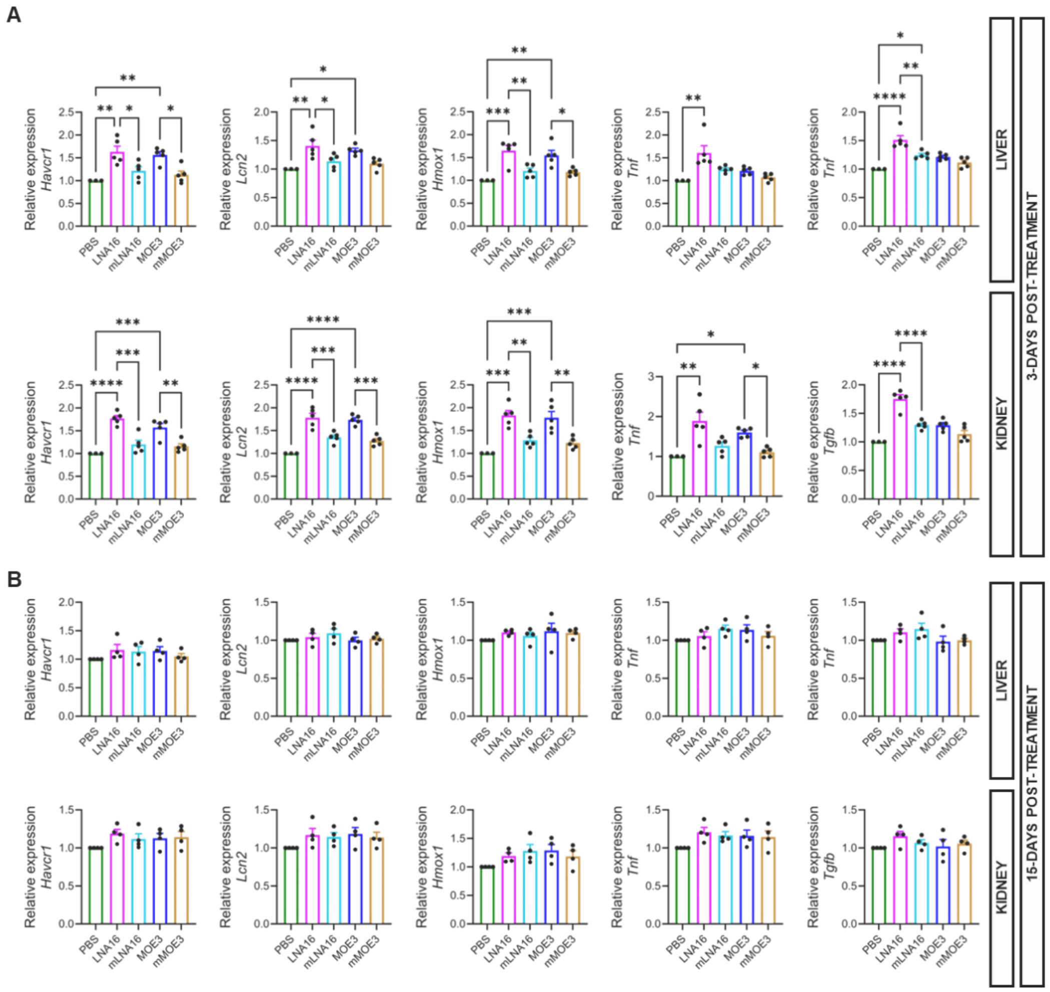

Figure S6: Hepatic and renal stress gene expression following gapmer administration.

(**A–B**) RT-qPCR quantification of a select panel of tissue stress markers, including *Havcr1*, *Lcn2*, *Hmox1*, *Tnf*, and *Tgfb* transcripts in liver and kidney tissues harvested at (**A**) 3 days and (**B**) 15 days post-treatment (11 mg/kg, retro-orbital). Wild-type B6 mice received retro-orbital (r.o.) administration of gapmers (11 mg/kg). Statistics, one-way ANOVA with Tukey's multiple comparisons test; **p*<0.05, ***p*<0.01, ****p*<0.001, *****p*<0.0001. Data are represented as mean ± standard error of the mean. Each black dot represents an individual biological replicate.
